# Inhibition of specific signaling pathways rather than epigenetic silencing of effector genes is the leading mechanism of innate tolerance

**DOI:** 10.3389/fimmu.2023.1006002

**Published:** 2023-01-26

**Authors:** Anna M. Masyutina, Polina V. Maximchik, Georgy Z. Chkadua, Mikhail V. Pashenkov

**Affiliations:** ^1^ Laboratory of Clinical Immunology, National Research Center “Institute of Immunology” of the Federal Medical-Biological Agency of Russia, Moscow, Russia; ^2^ Biological Faculty, Lomonosov Moscow State University, Moscow, Russia; ^3^ Faculty of Fundamental Medicine, Lomonosov Moscow State University, Moscow, Russia; ^4^ Laboratory of experimental diagnostics and biotherapy of tumors, N.N.Blokhin National Medical Research Center of Oncology of the Ministry of Health of the Russian Federation, Moscow, Russia

**Keywords:** innate immune response, tolerance, lipopolysaccharide, TLR4, muramyl peptides, NOD1, macrophages, transcriptome analysis

## Abstract

**Introduction:**

Macrophages activated through a pattern-recognition receptor (PRR) enter a transient state of tolerance characterized by diminished responsiveness to restimulation of the same receptor. Signaling-based and epigenetic mechanisms are invoked to explain this innate tolerance. However, these two groups of mechanisms should result in different outcomes. The epigenetic scenario (silencing of effector genes) predicts that activation of a PRR should broadly cross-tolerize to agonists of unrelated PRRs, whereas in the signaling-based scenario (inhibition of signaling pathways downstream of specific PRRs), cross-tolerization should occur only between agonists utilizing the same PRR and/or signaling pathway. Also, the so-called non-tolerizeable genes have been described, which acquire distinct epigenetic marks and increased responsiveness to rechallenge with the same agonist. The existence of such genes is well explained by epigenetic mechanisms but difficult to explain solely by signaling mechanisms.

**Methods:**

To evaluate contribution of signaling and epigenetic mechanisms to innate tolerance, we tolerized human macrophages with agonists of TLR4 or NOD1 receptors, which signal *via* distinct pathways, and assessed responses of tolerized cells to homologous restimulation and to cross-stimulation using different signaling, metabolic and transcriptomic read-outs. We developed a transcriptomics-based approach to distinguish responses to secondary stimulation from continuing responses to primary stimulation.

**Results:**

We found that macrophages tolerized with a NOD1 agonist lack responses to homologous restimulation, whereas LPS-tolerized macrophages partially retain the ability to activate NF-κB pathway upon LPS rechallenge, which allows to sustain low-level expression of a subset of pro-inflammatory genes. Contributing to LPS tolerance is blockade of signaling pathways required for IFN-β production, resulting in ‘pseudo-tolerization’ of IFN-regulated genes. Many genes in NOD1- or TLR4-tolerized macrophages are upregulated as the result of primary stimulation (due to continuing transcription and/or high mRNA stability), but do not respond to homologous restimulation. Hyperresponsiveness of genes to homologous rechallenge is a rare and inconsistent phenomenon. However, most genes that have become unresponsive to homologous stimuli show unchanged or elevated responses to agonists of PRRs signaling *via* distinct pathways.

**Discussion:**

Thus, inhibition of specific signaling pathways rather than epigenetic silencing is the dominant mechanism of innate tolerance.

## Introduction

Interaction of pathogen-derived molecules with pattern-recognition receptors (PRRs) of innate immune cells triggers expression of numerous genes coding for inflammatory and microbicidal effector molecules. Within a few hours after activation through a PRR, innate immune cells enter a transient state of tolerance, i.e. failure to produce pro-inflammatory cytokines upon restimulation of the same PRR (1–6). Despite suppressed cytokine responses, mice that have received a tolerizing dose of a PRR agonist show enhanced resistance to pathogens (7–9), indicating that tolerance is not unresponsiveness but altered responsiveness beneficial to the host. Therefore, induction of tolerance to specific PRR agonists may have a potential to prevent sepsis and other life-threatening conditions caused by excessive inflammatory responses to pathogens. At the same time, tolerance to a broad spectrum of PRR agonists may compromise host defence and lead to immunoparalysis observed at the late stage of bacterial sepsis (4, 10).

There is no consensus regarding mechanisms of innate tolerance. Most commonly, tolerance is thought of as a temporary post-activation block of signaling from a PRR, which can be mediated by multiple mechanisms, such as internalization or degradation of the receptor, removal of activating post-translational modifications from signaling proteins, or elevated expression of decoy molecules interrupting signaling cascades (2, 3, 5, 11). Alternatively, tolerance may be regulated epigenetically (12–14). In particular, it was noted that genes induced in murine macrophages by lipopolysaccharide (LPS), a Toll-like receptor 4 (TLR4) agonist, can be divided into two groups depending on their response to restimulation with LPS (12). The tolerizeable (T) genes, which include those coding for pro-inflammatory mediators, respond to restimulation substantially weaker than to primary stimulation or do not respond at all. The non-tolerizeable (NT) genes, which include genes coding for anti-microbial peptides, show equal or enhanced responses to restimulation as compared to primary stimulation. Differential behavior of T and NT genes in LPS-tolerized cells was suggested to depend on distinct modifications of chromatin in their promoters, resulting in either loss or gain of inducibility (12). Epigenetic mechanisms are also proposed to underlie innate immunological memory, i.e. long-term effects of PRR agonists which are a subject of intense investigation (15).

Both signaling-based and epigenetic scenarios of tolerance have strengths and weaknesses. The epigenetic scenario can explain the phenomenon of NT genes, although factors determining the choice between T and NT behavior have not been definitively established (4, 12, 14). Moreover, the dichotomy between T/pro-inflammatory and NT/antimicrobial genes is in line with enhanced antimicrobial protection of animals tolerized to PRR agonists, supporting the view of tolerance as a defense strategy based on minimizing inflammatory tissue damage along with boosting direct microbicidal mechanisms. However, if tolerance were regulated only epigenetically, then stimulation of any PRR would inhibit (cross-tolerize) transcriptional responses to subsequent stimulation of any other PRR, which contradicts to experimental data (4, 5). On the contrary, experimental observations concerning cross-tolerance are well explained by the signaling-based mechanisms. For example, cross-tolerance between pairs of TLRs occurs when both receptors utilize the same proximal adapter (e.g., MyD88), whereas usage of different adapters (MyD88 and TRIF) results in cross-priming rather than cross-tolerance (5). However, the signaling-based mechanisms do not explain the differential behavior of T and NT gene subsets, because both are regulated by apparently the same signaling pathways (12).

Besides TLRs, an important role in innate recognition of bacteria belongs to nucleotide-binding oligomerization domain 1 (NOD1) and NOD2, which sense fragments of bacterial peptidoglycan (muropeptides) (16, 17). Signaling pathways downstream of NOD1 and NOD2 are highly similar and rely on RIP2 kinase as the proximal adapter (18, 19). By contrast, TLR4 signals *via* MyD88 and TRIF adapters (20, 21). Thus, proximal parts of NOD1/NOD2- and TLR4-dependent signaling pathways are different, which should result in cross-priming (5). Indeed, LPS and NOD1/NOD2 agonists administered *in vivo* or added to cultures *in vitro* 6-24 h apart in any order usually prime to each other (4, 22–25). Nonetheless, some works have described inhibitory effects of NOD2 agonists on subsequent LPS-induced responses (26–28).

To address these controversies, we tolerized human macrophages *in vitro* by culturing them with a TLR4 agonist (LPS) or a NOD1 agonist (N-acetyl-D-muramyl-L-alanyl-D-isoglutamyl-meso-diaminopimelic acid [M-triDAP]) for 24 h, and assessed their signaling, transcriptional and metabolic responses to secondary stimulation with the same or with the other agonist. When interpreting responses of tolerized cells, two issues are important: (i) distinguishment between responses to secondary stimulation and continuing responses to primary stimulation; (ii) possible differences in kinetics of responses to primary and secondary stimulations. We developed a transcriptomics-based approach enabling to classify genes’ responses to secondary stimulation and distinguish them from continuing responses to primary stimulation. We show that vast majority of genes induced by TLR4 or NOD1 agonists in naïve macrophages do not respond to restimulation with the same agonist, and that the increased gene responsiveness to homologous restimulation is a rare and inconsistent phenomenon. However, genes not responding to homologous restimulation show unchanged or enhanced responsiveness to heterologous stimuli acting *via* distinct signaling pathways, indicating that epigenetic silencing is not the dominant mechanism of innate tolerance.

## Materials and methods

### Reagents

Synthetic M-triDAP, ultrapure LPS from E.coli O111:B4 and synthetic polyriboinosinic:polyribocytidilic acid (poly-I:C) were from *In vivo*gen (San Diego, CA). LPS was confirmed to be free of NOD1/NOD2 agonists using a test system described earlier (29). All agonists were dissolved in endotoxin-free water (*In vivo*gen). Complexes of poly-I:C with Lipofectamine 3000 (lipo-poly-I:C) were prepared as recommended by the manufacturer of Lipofectamine (Thermo Fisher Scientific, Waltham, MA). Recombinant human granulocyte-macrophage colony-stimulating factor (rhGM-CSF) was from Miltenyi Biotec (Bergisch Gladbach, Germany), and recombinant human interferon-beta (rhIFN-β1b) from Generium (Moscow, Russia). Complete culture medium (CCM) was RPMI-1640 supplemented with 2-mM L-glutamine and 10% fetal calf serum (all from Thermo Fisher Scientific). IκB kinase β inhibitor (PF-184) was from Tocris (Bristol, UK), actinomycin D (ActD) from Sigma (St-Louis, MO).

### Culture and stimulation of macrophages

**Figure 1:**
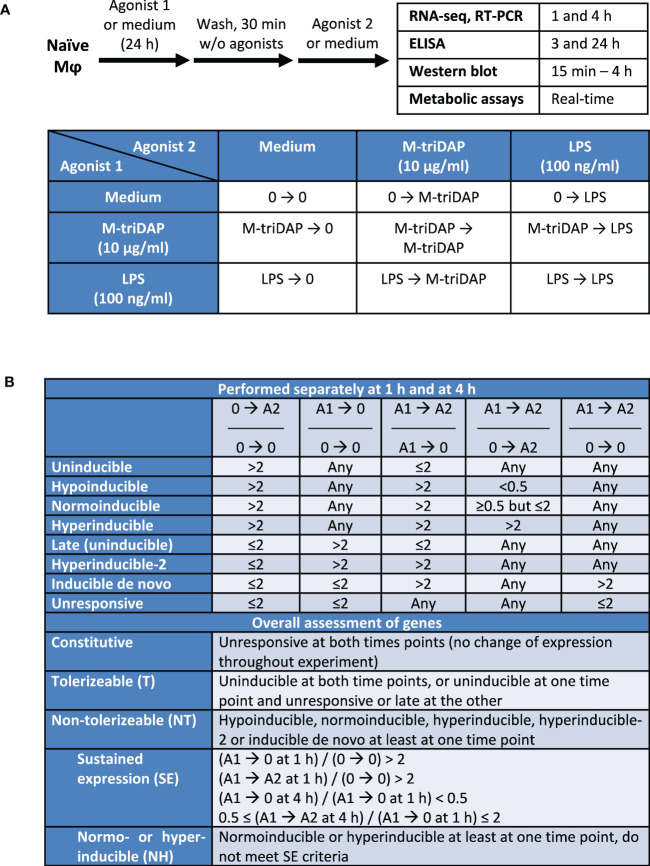
Experimental design of the study and criteria of gene classification. **(A)** Design of cell culture experiments. **(B)** Criteria of gene classification based on ratios of RPM. Agonist 1 (A1) is the one used for the 1^st^ stimulation (24 h), agonist 2 (A2) for the 2^nd^ stimulation (1 or 4 h). Agonists 1 and 2 can be the same. 0, culture without agonists.

Venous blood was collected from healthy donors 20-60 years old. Blood sampling was approved by the local Ethical Committee of the Institute of Immunology (protocol No 10/2017). Macrophages were obtained by culturing blood monocytes with rhGM-CSF (40 ng/ml) for 6 days as described (30). Before experiment, macrophages were replated in 24-well plates at 2*10^5^ cells/well in CCM without rhGM-CSF, and allowed to rest for 24 h. To induce tolerance, macrophages were cultured for 24 h with M-triDAP (10 µg/ml), LPS (100 ng/ml) or without agonists as a control. After 24 h, media were removed, cells were gently washed with warm RPMI-1640, wells were filled with fresh warm CCM, and cells were allowed to rest for 30 min. Then, medium, M-triDAP or LPS at the same concentrations as above were added so as to produce all combinations of the 1^st^ and the 2^nd^ stimulus (see **Figure 1A** for experimental design). In some experiments, naïve and LPS-tolerized macrophages were cultured with rhIFN-β1b (10 U/ml) or lipo-poly-I:C (equivalent of 1 μg/ml poly-I:C). Enzyme inhibitors were added 15 min prior to 2^nd^ stimulation. Cell lysates for RNA isolation and Western blotting as well as cell culture supernatants were collected at indicated time points and stored at –70°C.

### Next-generation RNA sequencing

We performed two independent, identically designed experiments (**Figure 1A**) using macrophages from the same donor generated at two different occasions (two biological replicates). To assess kinetics of gene expression, samples were obtained 1 h and 4 h after 2^nd^ stimulation, which yielded sets of 18 samples from each experiment. Total RNA was extracted using a kit from Jena Bioscience (Jena, Germany, cat# PP-210) and treated with DNase I (NEB, Ipswich, MA, cat# M0303). mRNA was purified using oligo-dT-coated superparamagnetic beads (NEB, cat# E7490). Barcoded cDNA libraries were prepared using NEBNext^®^ Ultra™ II Directional RNA Library Prep with Sample Purification Beads (NEB, cat# E7765) and NEBNext^®^ Multiplex Oligos for Illumina^®^ (NEB, cat# E7335 and E7500) according to manufacturer’s instructions. Quality of libraries was assessed using 2100 Bioanalyzer system (Agilent Technologies, Santa Clara, CA). Pools of 18 cDNA libraries were sequenced in two independent runs using Illumina NovaSeq 6000 platform (Evrogen, Moscow, Russia), yielding 27.7 ± 3.7*10^6^ and 23.4 ± 4.5*10^6^ reads per library in the 1^st^ and the 2^nd^ experiment, respectively. FASTQ files were generated using bcl2fastq v2.20 Conversion Software (Illumina). Reads were aligned against reference human transcriptome (ftp://ftp.ensembl.org/pub/release-98/fasta/homo_sapiens/cdna/Homo_sapiens.GRCh38.cdna.abinitio.fa.gz) using Salmon software package (31). Percentages of successfully aligned reads were 90.2 ± 3.98% in the 1^st^ experiment and 91.4 ± 0.57% in the 2^nd^ experiment. Counts for different mRNA variants from the same gene were summed up.

Genes were selected for further analysis according to abundance of their transcripts (at least 50 raw counts in at least one library in each of the two experiments) and function (only protein-coding genes), which produced a set of 11,964 genes. Global expression profiles of synonymous libraries from two experiments showed strong correlations (**Supplementary Figure S1A**). Measurements of individual genes’ expression from two RNA-seq experiments also strongly correlated, especially in case of genes with most variable expression (**Supplementary Figures S1B, C**).

Read-per-million (RPM) values were used to analyse differential gene expression. More than two-times differences of RPMs were considered biologically significant. To avoid division by zero, counts equal to zero were replaced with ones prior to RPM calculation. RNA-seq data were deposited in GEO, accession number GSE207510.

### Classification of genes according to their behavior after 1st and 2nd stimulation

Classifications were performed separately for each stimulation sequence (LPS➔LPS, M-triDAP ➔ M-triDAP, LPS ➔ M-triDAP, M-triDAP ➔ LPS) in each RNA-seq experiment, results of synonymous classifications from two experiments were compared (see below).

Within each classification, two subclassifications were initially constructed (separately for time points 1 h and 4 h after 2^nd^ stimulation), based on 5 indices shown in **Figure 1B** (upper part). Unlike previous works (4, 12), we compared expression of genes after secondary stimulation (agonist 1 ➔ agonist 2) not only to their expression after primary stimulation (0 ➔ agonist 2), but also to expression in tolerized macrophages recultured without agonists for the same time (agonist 1 ➔ 0), which allowed to distinguish responses to secondary stimulation from continuing responses to primary stimulation. We first identified inducible genes whose expression after 1 or 4 h of primary stimulation (0 ➔ agonist 2) was increased more than twice compared to expression in unstimulated macrophages. Depending on their inducibility after secondary stimulation (agonist 1 ➔ agonist 2), these genes were further classified into uninducible, hypoinducible, normoinducible and hyperinducible. Uninducible genes did not respond to secondary stimulation, i.e. were not induced above the levels attained in agonist 1 ➔ 0 cultures. Hypo-, normo- and hyperinducible genes responded to secondary stimulation weaker, equal or stronger, respectively, than to primary stimulation. Many genes, which we call ‘late’, were not induced after 1 or 4 h of primary stimulation, but were upregulated after 24 + 1 or 24 + 4 h, i.e. in cultures agonist 1 ➔ 0. Most of these late genes did not respond to secondary stimulation (similar to uninducible genes, albeit it should be noted that responses of these genes can be correctly assessed after another 24 + 1 or 24 + 4 h, which was not done here). However, some of the late genes were further upregulated (more than twice) even after 1 or 4 h of secondary stimulation, and were termed ‘hyperinducible-2’. Genes that were not induced after primary stimulation and reculture without agonists, but induced after secondary stimulation (in cultures agonist 1 ➔ agonist 2) were classified as inducible *de novo*. Finally, genes that were expressed but did not respond to either primary or secondary stimulation were termed unresponsive (**Figure 1B**). This approach allowed to classify >98% of all analysed genes.

Based on subclassifications constructed at 1 and 4 h, genes were given an overall assessment to characterize their behavior upon the entire stimulation sequence (**Figure 1B**, lower part). A gene was considered tolerizable (T) if classified as uninducible at both time points, or as uninducible at one time point and unresponsive or late at the other. A gene was considered non-tolerizable (NT) if classified as hypoinducible, normoinducible, hyperinducible, hyperinducible-2 or inducible *de novo* at least at one time point, irrespectively of response at the other time point. This definition of NT genes included all genes showing any response to secondary stimulation, not necessarily stronger than to the primary stimulation. Within the NT category, some genes fell into one of two subcategories: genes with sustained expression (SE), and normo- or hyperinducible (NH) genes (**Figure 1B**). SE genes met following criteria: (i) after the 24-hour culture with agonist 1 and 1-hour reculture with or without agonist 2, expression of the gene is >2 times higher than in unstimulated cells; (ii) after 4-hour reculture without agonists (agonist 1 ➔ 0 at 4 h), expression falls >2 times compared to 1-hour reculture without agonist; (iii) after 4-h reculture with agonist (agonist 1 ➔ agonist 2 at 4 h), expression is changed <2 times (sustained) as compared to agonist 1 ➔ 0 at 1 h. A gene was classified as NH if it was normoinducible or hyperinducible at least at one time point and did not meet SE criteria.

To assess reproducibility of the observed patterns of gene behavior in the two RNA-seq experiments, we used χ^2^ test as follows. Suppose experiment 1 and experiment 2 produced gene sets S_1_ and S_2_, comprising, respectively, n_1_ and n_2_ genes of the given class. The intersection between S_1_ and S_2_ is an S_3_ set comprising n_3_ genes. The total number of analysed genes was 11,964 (see above). The probability that members of S_1_ and S_2_ coincide randomly is p_12_ = (n_1_*n_2_)/11964^2^, and the number of genes in case of random coincidence will be n_12_ = p_12_*11964 = (n_1_*n_2_)/11964, rounded to the nearest integer. In case n_3_ > n_12_, proportions of expected (n_12_) and actual (n_3_) coincidences were compared by χ^2^ test. If p < 0.05 was obtained, behavior of S_3_ genes was considered nonrandom (reproducible). To confirm RNA-seq results, expression of selected genes was also analysed by semi-quantitative RT-PCR in a larger number of experiments, applying the same classification criteria as used for RNA-seq data.

### Further analysis of RNA-seq data

Gene set enrichment analysis (GSEA) was performed using a software developed by UC San Diego and Broad Institute (32). Cluster analysis and heat maps were done using Morpheus (https://software.broadinstitute.org/morpheus ).

Transcription factor binding site (TFBS) predictions were done using CiiiDER software, which uses MATCH algorithm and JASPAR position frequency matrices (33). Minimum allowed similarity score between potential TFBS and ‘ideal’ TFBS sequence was 0.85 (score 0 denotes complete mismatch, score 1 is exact match). TFBS enrichment scores (ES) were calculated as follows: ES = log_2_((n_1_+0.5)/(N_1_+0.5)) – log_2_((n_2_+0.5)/(N_2_+0.5)), where n_1_ and n_2_ are numbers of genes containing a TFBS in gene sets 1 and 2, N_1_ and N_2_ are total number of genes in sets 1 and 2 (33). Significance of enrichment was assessed by χ^2^ test. The software automatically chose a cut-off (in the range of 0.85-1) at which the difference between gene sets 1 and 2 was most significant.

### Real-time PCR

0.5 µg total RNA was reverse-transcribed by RevertAid™ first strand cDNA synthesis kit using oligo-dT primer (Thermo Fisher Scientific). We assessed expression of 23 genes from the following categories: cytokines (*IFNB1, IL1B, IL6, IL10, IL12B, IL23A, TNF*), interferon response markers (*MX1*), NOD1 receptor (*NOD1*) and its adapter (*RIPK2 [RIP2]*), NF-κB family transcription factors (TFs) (*NFKB1 [NFKB-p50], RELA [p65]*), negative regulators of NF-κB signaling (*NFKBIA [IKBA], TNFAIP3 [A20]*), NT genes from (13) (*FPR1, PTGES*), E2F family TFs (*E2F1, E2F2*), and several genes classified as hyperinducible upon LPS ➔ LPS sequence in our RNA-seq experiments (*WNT5A, PIM2, PKIG, RIPOR2*). Amplifications were performed using 7300 Real Time PCR System (Applied Biosystems) under conditions described earlier (30, 34). Sequences of primers are listed in **Supplementary Table S1**. Relative expression (RE) was calculated by the 2^–ΔΔCt^ method using non-stimulated macrophages from each donor as reference samples and *GAPDH* as the house-keeping gene. Two-times or higher differences of RE were considered biologically significant.

### Assessment of mRNA stability

Macrophages were subjected to 1^st^ and 2^nd^ stimulations as described above. ActD (10 µg/ml) was added at 1 h of 2^nd^ stimulation. RNA samples were obtained at addition and 1 h after addition of ActD. RT-PCR was performed as above. Rates of mRNA decay were calculated as ratios of mRNA levels after 1 h with ActD to levels before addition of ActD.

### Isolation and analysis of nascent transcripts

The procedure was adapted from (35) and (36). Macrophage monolayers (approximately 10^6^ cells/sample) were rinsed with ice-cold PBS and incubated for 12 min with an ice-cold hypotonic buffer (10 mM HEPES, 10 mM KCl, 1,5 mM MgCl_2_, pH 7.9) supplemented with protease inhibitor cocktail (cOmplete Mini, Thermo Scientific). Cytoplasmic membranes were lysed by adding Nonidet P-40 to a final concentration of 0.6% (30 s) followed by vigorous pipetting. Lysates were centrifuged for 5 min at 2000 g, 4°С. Supernatants (cytosolic fraction) were removed and stored at –70°C, while nuclear pellets were resuspended in nuclear wash buffer (10 mM HEPES, 250 mM sucrose, 1 mM DTT, 1x protease inhibitor cocktail, 50 U/ml RiboLock [Thermo Scientific], рН 7.9) and centrifuged as above. Nuclear pellets were treated with a urea buffer (1 M urea, 20 mM HEPES, 7.5 mM MgCl_2_, 0.2 mM EDTA, 300 mM NaCl, 1% Nonindet P-40, 50 U/ml RiboLock) for 3 min at 4°С and centrifuged 3 min at 5000 g. Supernatants containing nucleoplasm and RNA species weakly bound to chromatin were removed, and the pellet was washed once again with urea buffer (3 min at 5000 g, 4°C). The washed chromatin pellet was dissolved in TriReagent (Sigma) at 50°C (5 min). Chromatin-associated RNA (caRNA) was isolated as recommended by the manufacturer of TriReagent and dissolved in RNase-free water. RNA samples were treated with DNase (NEB, cat# M303) and cleaned using Monarch RNA Cleanup Kit Protocol (NEB, cat# T2030). Purified caRNA was reverse-transcribed by RevertAid RT Kit using random hexamer primer (Thermo Scientific). To capture unspliced and partially spliced full-length pre-mRNA as well as mature mRNA, PCR primers were placed in the last exons of genes analysed (**Supplementary Table S2**). RT-PCR was performed as described above. Levels of U6 small nuclear RNA were used for normalization.

### ELISA

Levels of TNF and IL-6 in cell culture supernatants were determined by sandwich ELISA (eBioscience/Thermo Fisher Scientific).

### Western blotting

The procedure was described earlier (30, 34). Blots were probed with rabbit antibodies against IκBα (cat# 9242), RIP2 (cat# 4142), phospho-p38 (pT180/pY182, cat# 9211), phospho-JNK (pT183/pY185, cat# 4668) or total p38 (cat# 9212), or with mouse mAbs against phospho-ERK1/2 (pT202/pY204, cat# 9106) or total ERK1/2 (cat# 4696), all from Cell Signaling Technologies (Danvers, MA). Staining for α-tubulin (clone DM1A, Novus Biologicals, Centennial, CO) was used as a loading control.

### Real-time analysis of cell metabolism

Extracellular acidification rate (ECAR) and oxygen consumption rate (OCR) were measured using Seahorse XFe96 Analyzer (Agilent Technologies) as described earlier (34), with modifications. Briefly, macrophages were trypsinized, counted, and seeded into Seahorse XF96 plates (Agilent Technologies) at 16,000 cells per 80 µl CCM per well. Cells were allowed to adhere, whereafter agonists or medium were added for 24 h. Next day, cells were washed twice with 160 µl XF base medium (Agilent Technologies) supplemented with 2 mM L-glutamine, 11 mM D-glucose (Sigma) and 10% fetal calf serum. After washes, wells were filled with 80 µl of the above medium and pH-equilibrated in an atmospheric air incubator for 1 h, whereafter plates were transferred into the analyzer. ECAR and OCR were measured every 9 min. Three basal measurements were taken, then agonists or medium were injected and a further 22 measurements were performed.

### Statistics

Proportions were compared by χ^2^ test. Correlations were analysed by Pearson’s test. Multiple comparisons were performed by repeated-measures ANOVA with Tukey’s correction.

## Results

### Transcriptional responses of naïve macrophages to M-triDAP and LPS

Treatment of naïve macrophages with M-triDAP or LPS induced expression of hundreds of genes coding for inflammatory mediators, interferon response proteins, microbicidal proteins (**Supplementary Figures S2A, S2B**). Expression of many genes upregulated at 4 h of primary stimulation remained stable or further increased at 24 + 1 h (in cultures treated with agonists for 24 h and recultured without agonists for 1 h) (**Supplementary Figure S2C**). Another large subset of genes was not induced at 1 or 4 h of primary stimulation but upregulated at 24 + 1 h (late genes) (**Supplementary Figure S2C**). Thus, by 24 h after primary stimulation, the time point when tolerance is usually assessed, there is an ongoing transcriptional response to primary M-triDAP or LPS stimulation.

### Responses of LPS-tolerized macrophages to LPS restimulation

**Figure 2:**
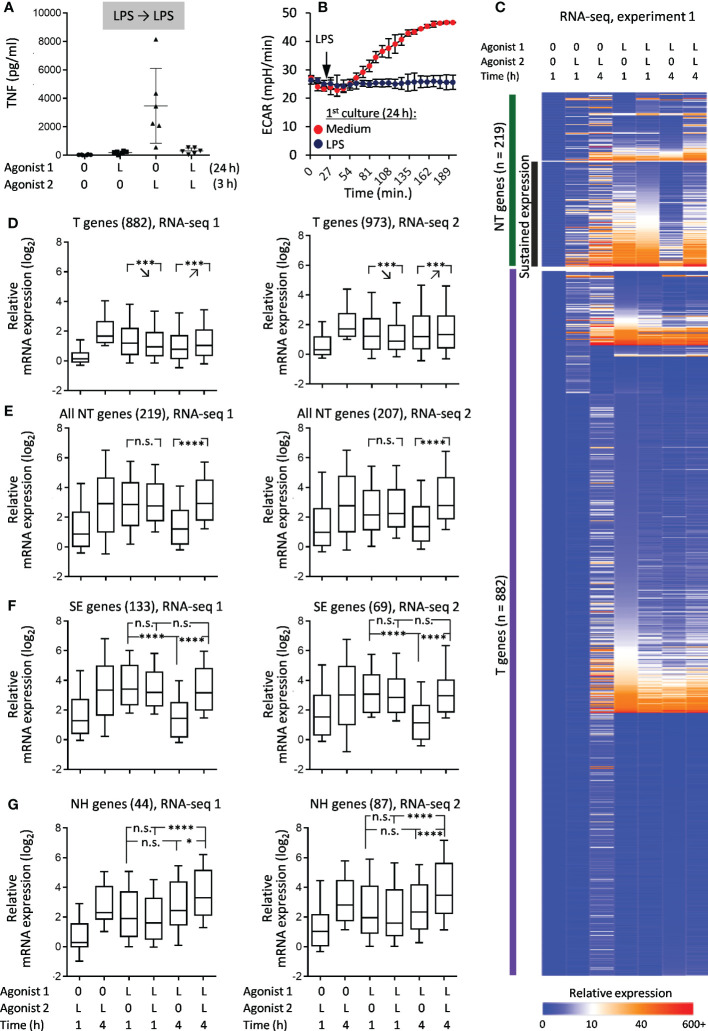
Responses of LPS-tolerized macrophages to LPS restimulation. Macrophages were cultured for 24 h with LPS (L) or medium alone (0), washed and recultured with LPS or medium alone for indicated periods of time. **(A)** Levels of TNF in supernatants of naïve and LPS-tolerized macrophages (ELISA, 6 experiments, M±σ). **(B)** Kinetics of ECAR of naïve and LPS-tolerized macrophages after addition of LPS (1 representative experiment out of 6, M±σ of quadruplicate wells). **(C)** Heat map of relative gene expression (RPM_sample_/RPM_baseline_) in RNA-seq experiment 1. **(D–G)** Statistics of relative gene expression (log_2_-transformed) of T genes **(D)**, all NT genes **(E)**, SE genes **(F)**, NH genes **(G)** in RNA-seq experiments 1 and 2. Boxes represent 25^th^ and 75^th^ percentiles, lines within boxes are medians, whiskers are 10^th^ and 90^th^ percentiles. P-values by repeated measures ANOVA with Tukey correction (*p < 0.05, ***p < 0.001, ****p < 0.0001, n.s. = non-significant).

Tolerance was initially assessed using two parameters: TNF production (depends on *de-novo* gene expression) and activation-induced boost of glycolysis (does not require *de-novo* gene expression (37)). In macrophages treated with LPS for 24 h and restimulated with LPS, both types of responses were strongly inhibited as compared to LPS responses of naïve macrophages (**Figures 2A, B**), confirming the state of LPS tolerance. RNA-seq showed that most genes in LPS-tolerized macrophages did not respond to LPS restimulation (LPS➔LPS), which placed them in the T category (**Figures 2C, D**). However, a significant subset of genes showed some response to restimulation (NT pattern) (**Figures 2C, E**). Many of the NT genes fulfilled criteria for sustained expression (SE): levels of their mRNA during 2^nd^ LPS stimulation were sustained at the level achieved after 1^st^ stimulation, but decreased more than twice after 4 hours of reculture without LPS (**Figure 2F**). However, only a small minority of NT genes were NH, i.e. responded to secondary stimulation equally or greater than to primary stimulation and did not meet SE criteria (**Figure 2G**).

**Figure 3:**
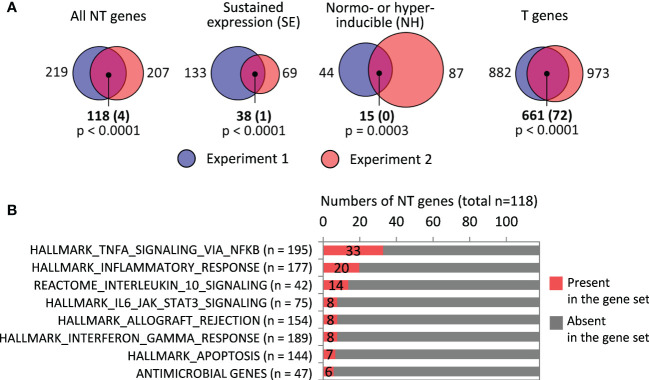
Analysis of responses of LPS-tolerized macrophages to LPS restimulation. **(A)** Sizes of T, NT, SE and NH gene sets identified in RNA-seq experiments 1 and 2, and their intersections (numbers of genes). Shown in parentheses are expected numbers of coincidences if they were purely random. Differences between expected and actual numbers of coincidences were assessed using χ^2^ test. **(B)** Gene sets significantly over-represented among the 118 NT genes (all with p < 0.001 in χ^2^ test).

Overlap between T, NT, SE and NH gene sets from two RNA-seq experiments was significantly greater than expected by chance (**Figure 3A**).

In an earlier transcriptomic profiling of LPS-tolerized murine macrophages, NT genes were defined as those whose expression after secondary LPS stimulation was equal or greater than after primary stimulation (Expression_LPS➔LPS_/Expression_0➔LPS_ ≥ 1, assessed at a single point of 4 h) (12). Although this definition is supposed to capture genes with the same characteristics as our NH genes, it does not take into account residual gene expression after primary stimulation (LPS➔0) or kinetics of gene expression. When we defined NT genes as in (12), we obtained 271 and 360 NT genes in experiments 1 and 2, respectively, which is 4-8 times greater than the number of NH genes identified in our study. Thus, the criterium used in (12) overestimates the number of genes with unchanged or enhanced responsiveness to secondary LPS stimulation.

Functionally, many of the NT genes defined according to our criteria encode pro-inflammatory proteins (**Figure 3B**, **Supplementary Figure S3**), although the expression of pro-inflammatory genes in LPS-tolerized cells is several orders lower than peak levels during primary stimulation (**Supplementary Figure S4A**). Only a few NT genes code for antimicrobial peptides/proteins (**Figure 3B**, **Supplementary Figure S3**).

Response patterns of 20 selected genes were examined by real-time PCR (RT-PCR) in additional experiments using macrophages from independent donors (**Supplementary Figures S4A, B**). Although responses of individual genes were not uniform, RNA-seq- and RT-PCR-based classifications generally corresponded to each other: (i) SE genes identified in RNA-seq experiments (*IL1B, IL6, IL12A, IL23A, TNF, RIPK2, NFKB1*) showed mainly SE or, more seldom, T behavior in RT-PCR experiments; (ii) T genes identified by RNA-seq (*IL10, IFNB1, MX1, NOD1, RELA, NFKBIA, FPR1, PTGES*) showed mostly T or, more seldom, constitutive behavior in RT-PCR. *FPR1* and *PTGES* genes, which had been classified as NT in an earlier study (12), showed a high residual expression after 1^st^ LPS stimulation and did not respond to 2^nd^ stimulation in most donors, which placed them in the T category. Finally, genes identified as NH in RNA-seq experiments showed a very variable behavior in RT-PCR experiments; importantly, NH pattern was observed in only 3/10, 1/10, 0/10 and 4/10 donors for *PIM2, PKIG, RIPOR2, WNT5A* genes, respectively, indicating that their NH behavior may be donor-specific.

**Figure 4:**
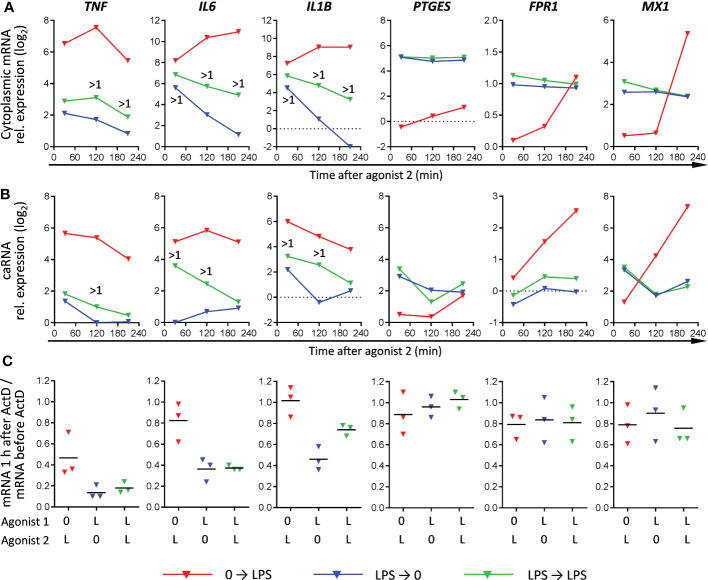
Relationship between mRNA expression patterns, mRNA transcription and mRNA stability in naïve and LPS-tolerized macrophages (RT-PCR). Cells were cultured 24 h without or with LPS, washed and recultured without or with LPS for indicated periods of time. **(A, B)** Kinetics of cytoplasmic mature mRNA expression and chromatin-associated RNA (caRNA) expression, respectively, in naïve and LPS-tolerized macrophages. One experiment out of two with similar results. >1, more than 1 log_2_ difference between LPS➔0 and LPS➔LPS macrophages. Log_2_ mRNA or caRNA expression in untreated macrophages equals zero. **(C)** stability of mature cytoplasmic mRNA in naïve macrophages stimulated by LPS and in LPS-tolerized macrophages recultured with or without LPS (3 experiments, bars denote means). Actinomycin D (ActD) was added at 1 h of (re)stimulation and cells were further cultured for 1 h. Results are expressed as ratios of mRNA levels after and before addition of ActD.

As can be seen from RNA-seq and RT-PCR data (**Figures 2C, D**, **Supplementary Figure S4**), many T genes, such as *PTGES*, *FPR1* and *MX1*, had a stably elevated residual mRNA expression after the 1^st^ LPS stimulation, but did not respond to the 2^nd^ stimulation. To verify lack of their response to 2^nd^ LPS stimulation, we assessed levels of their nascent chromatin-associated transcripts in parallel with mature cytoplasmic mRNA. We confirmed the T behavior of *PTGES*, *FPR1* and *MX1* (no difference in nascent or mature transcript levels between LPS➔0 and LPS➔ stimulations) (**Figures 4A, B**). At the same time, *PTGES*, *FPR1* and *MX1* mRNAs showed a high stability in either LPS➔0 or LPS➔ conditions (**Figure 4C**), which explains their stably elevated levels after removal of LPS from the medium. By contrast, *IL1B*, *IL6* and *TNF* genes responded to 2^nd^ LPS stimulation (>1 log_2_ higher nascent and mature transcript levels in LPS➔LPS compared to LPS➔0 conditions) (**Figure 4A, B**), contributing to sustained expression of their mRNAs in LPS➔LPS conditions. However, *IL1B*, *IL6* and *TNF* mRNAs were relatively unstable in LPS➔0 conditions (**Figure 4C**), explaining their rapid drop after removal of LPS from the medium (LPS➔0).

In addition, we specifically analysed genes that were induced in naïve macrophages at 1 or 4 h of LPS treatment but returned to baseline expression after 24 + 1 h (LPS➔0). 429 and 461 such genes were found in RNA-seq experiments 1 and 2, respectively. Among them, 23 and 33 NT genes, respectively, were identified; however, only two of these NT genes were in common between the experiments, which was not significantly greater than expected by chance.

### Responses of M-triDAP-tolerized macrophages to M-triDAP restimulation

**Figure 5:**
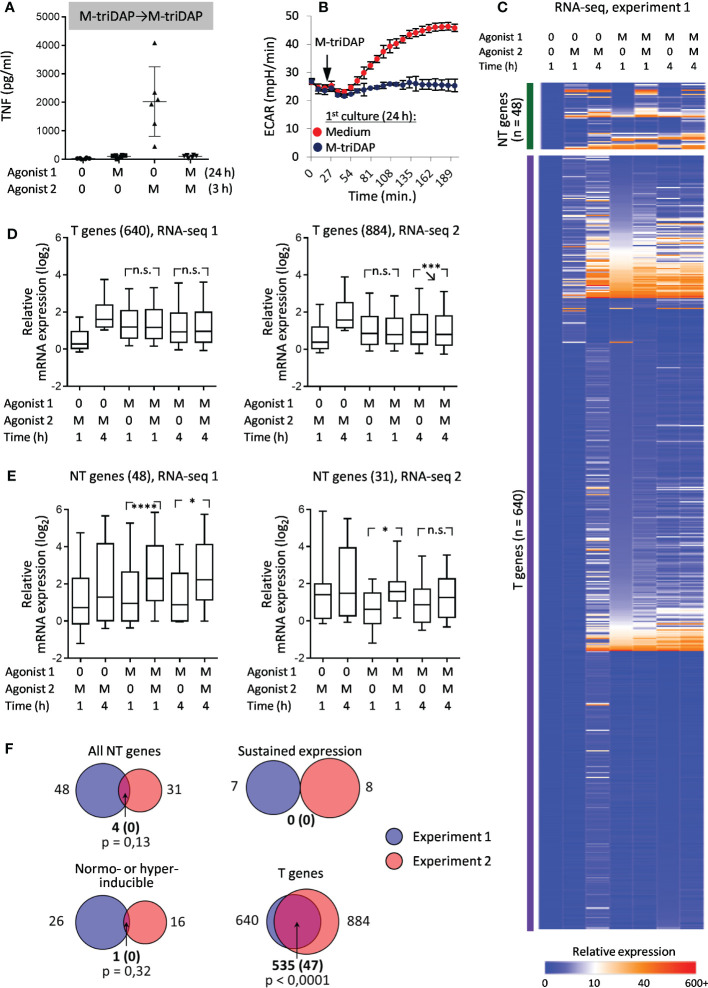
Responses of M-triDAP-tolerized macrophages to M-triDAP restimulation. Macrophages were cultured for 24 h with M-triDAP (M) or medium alone (0), washed and recultured without or with M-triDAP for indicated periods of time. **(A)** Levels of TNF in supernatants of naïve and M-triDAP-tolerized macrophages after restimulation with M-triDAP (ELISA, 6 experiments, M±σ). **(B)** Kinetics of ECAR of naïve and M-triDAP-tolerized macrophages after addition of M-triDAP (1 representative experiment out of 6, M±σ of quadruplicate wells). **(C)** Heat map of relative gene expression (RPM_sample_/RPM_baseline_) in RNA-seq experiment 1. **(D, E)** Statistics of relative gene expression (log_2_-transformed) of T genes **(D)** and all NT genes **(E)** in RNA-seq experiments 1 and 2. **(F)** Sizes of T, NT, SE and NH gene sets identified in RNA-seq experiments 1 and 2, and their intersections (numbers of genes). Shown in parentheses are expected numbers of coincidences if they were purely random. Statistical analysis as in **Figures 2** and **3**. *p < 0.05, ***p < 0.001, ****p < 0.0001, n.s. = non-significant.

Pre-treatment of macrophages with M-triDAP inhibited TNF production and activation-induced increase of ECAR glycolysis in response to rechallenge with M-triDAP (**Figures 5A, B**), similar to what was observed with the LPS➔LPS sequence. However, in M-triDAP-tolerized macrophages much fewer genes were classified as NT (**Figures 5C-E**). Only four genes were classified as NT in both RNA-seq experiments, which was not significantly greater than expected by chance (**Figure 5F**). Only one gene satisfied NH criteria in both experiments and none showed an SE behavior (**Figure 5F**). RT-PCR confirmed lack of transcriptional response to secondary M-triDAP stimulation, with vast majority of analysed genes classified as T or constitutive (**Supplementary Figures S5A, B**).

**Figure 6:**
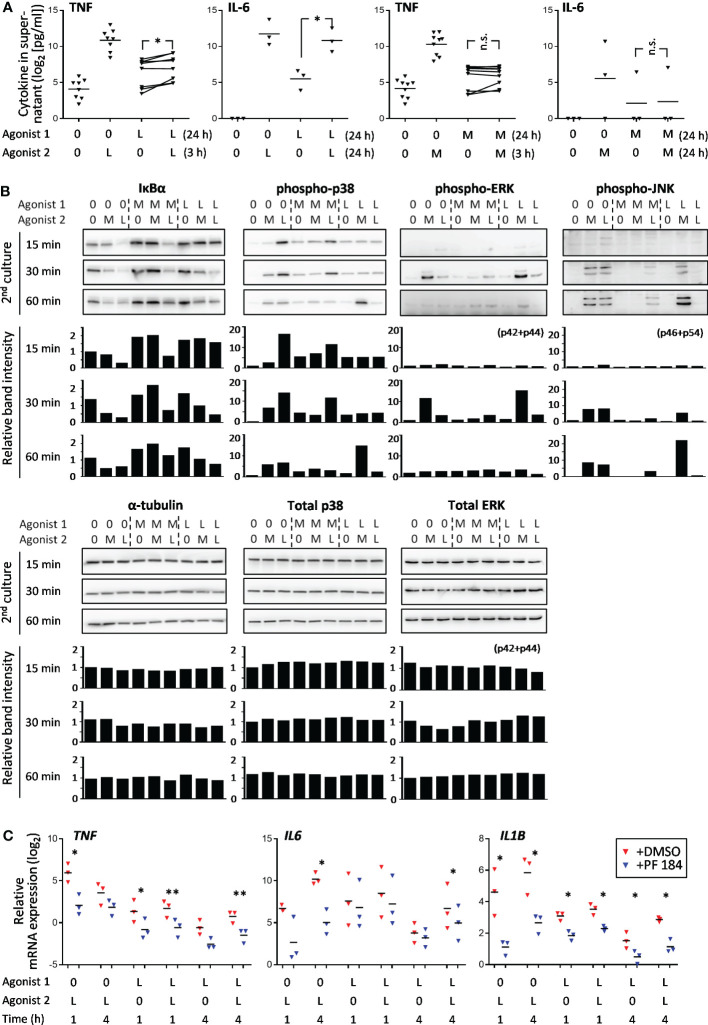
Activation of NF-κB and p38 pathways in naïve, M-triDAP- and LPS-tolerized macrophages after their reculture with medium (0), M-triDAP (M) or LPS (L). **(A)** Levels of TNF and IL-6 in macrophage supernatants after indicated stimulation sequences (3 to 9 experiments). Statistical analysis by repeated measures ANOVA with Tukey correction. * p < 0.05, n.s. = non-significant. **(B)** Immunoblotting for IκBα, phospho-p38, phospho-ERK1/2, phospho-JNK, total p38, total ERK1/2 and α-tubulin at indicated time points after 2^nd^ stimulation (one representative Western blot out of three) and corresponding densitometry data normalized to untreated macrophages at 15 min. **(C)** Effect of IκB kinase inhibitor on *TNF*, *IL6* and *IL1B* mRNA expression in naïve and LPS-tolerized macrophages (RT-PCR). Cells were cultured without or with LPS for 24 h, washed, incubated without or with PF 184 (5 μM) for 15 min, whereafter recultured without or with LPS for 1 or 4 h. 3 experiments, bars denote means. *p < 0.05, **p < 0.01 for comparisons between cells treated or not with PF 184 in identical stimulation conditions (paired t-test).

The SE behavior of *TNF* and *IL6* gene in LPS➔LPS settings and lack thereof in M-triDAP➔M-triDAP settings was confirmed at the protein level: LPS➔LPS sequence induced a significant increase in secreted TNF and IL-6 levels as compared to LPS➔0 conditions, whereas M-triDAP➔M-triDAP sequence did not augment cytokine production as compared to M-triDAP➔0 (**Figure 6A**).

Thus, at the transcriptional level, M-triDAP-tolerized macrophages lose responsiveness to the same agonist, whereas LPS-tolerized macrophages remain partially responsive to LPS. This behavior of LPS-tolerized cells is not due to non-optimal tolerization conditions, because sufficiently high concentration of LPS was used (100 ng/ml), and changing the duration of primary LPS stimulation to 18 or 30 h did not alter patterns of *TNF* and *IL6* mRNA expression (data not shown). The phenomenon of preserved or increased gene inducibility in LPS-tolerized macrophages upon LPS restimulation is inconsistent. However, LPS restimulation can sustain elevated residual expression of some genes, which is rapidly lost in the absence of LPS.

### The NT behavior of genes correlates with activation of signaling pathways in tolerized cells

The relatively large number of NT genes observed upon LPS➔LPS stimulation suggests that TLR4-dependent signaling pathways remain partially active in LPS-tolerized macrophages. We assessed NF-κB and MAP kinase pathways downstream of TLR4 and NOD1 in naïve and tolerized cells (**Figure 6B**). In naïve macrophages, as expected, M-triDAP and LPS induced rapid IκBα degradation as well as p38, ERK and JNK phosphorylation. In M-triDAP- or LPS-tolerized macrophages recultured with medium, levels of IκBα were increased compared to baseline. No IκBα degradation was observed in M-triDAP-tolerized cells upon M-triDAP restimulation, whereas significant IκBα degradation occured in LPS-tolerized macrophages restimulated with LPS, although with a delay compared to naïve LPS-stimulated macrophages (**Figure 6B**). This indicated activation of NF-κB pathway in LPS-tolerized but not M-triDAP-tolerized macrophages upon homologous restimulation. Pharmacological inhibition of IKKβ resulted in a significant decrease of *IL1B*, *IL6* and *TNF* mRNA levels in either naïve or LPS-tolerized macrophages (re)stimulated with LPS, confirming functional activity of this pathway (**Figure 6C**). Levels of phospho-p38 were increased in M-triDAP- or LPS-tolerized cells, but no additional increase upon homologous restimulation was observed (**Figure 6B**). JNK phosphorylation was completely blocked in homologous restimulation settings, while a minor transient increase in phospho-ERK was observed in LPS-tolerized cells restimulated with LPS. Levels of total p38 and ERK1/2 were not significantly affected in any condition tested (**Figure 6B**). Thus, partial transcriptional responsiveness of LPS-tolerized macrophages to homologous restimulation and lack thereof in M-triDAP-tolerized macrophages correlates with activation of the NF-κB pathway.

### Responses of M-triDAP- and LPS-tolerized macrophages to cross-stimulation

If T genes in tolerized macrophages were silenced at the transcriptional level, they would lose responsiveness not only to the same agonist, but also to agonists of other PRRs. To test this, we tolerized macrophages with LPS and restimulated with M-triDAP, or vice versa. To allow correct comparisons between different stimulation sequences, we selected 446 genes that were inducible in naïve macrophages by short-term (1 or 4 h) treatment both with M-triDAP and with LPS in both RNA-seq experiments.

**Figure 7:**
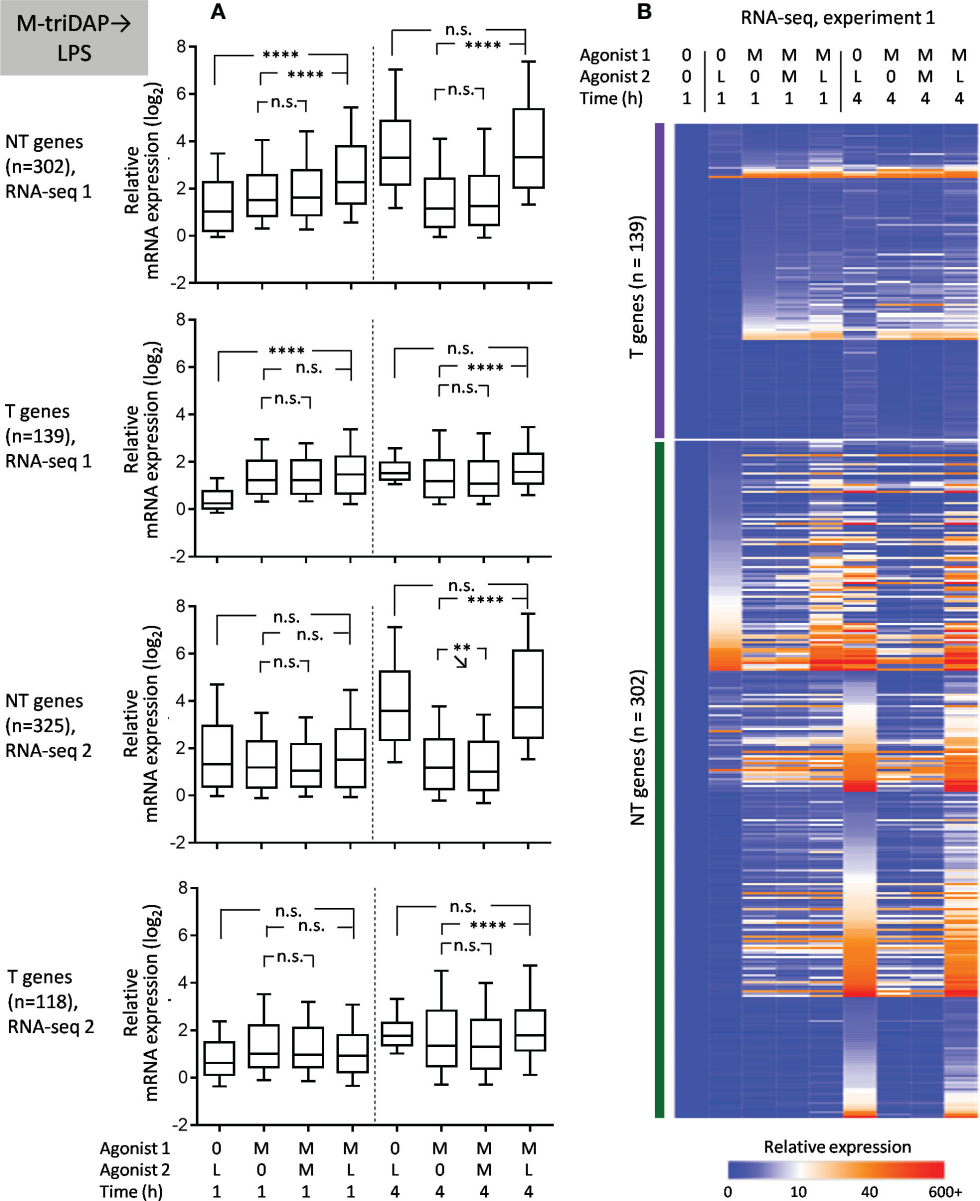
Responses of macrophages tolerized with M-triDAP (M) to stimulation with LPS (L). Only genes inducible in naïve macrophages both by M-triDAP and by LPS in both experiments after 1 and/or 4 h of stimulation are included (n = 446). **(A)** General statistics of T and NT gene expression in RNA-seq experiments 1 and 2 upon M-triDAP➔LPS stimulation. Responses of naïve macrophages to LPS (0➔LPS), of M-triDAP-tolerized macrophages to M-triDAP (M-triDAP➔M-triDAP) as well as residual M-triDAP-induced expression (M-triDAP➔0) are shown in parallel. **(B)** Heat map of relative gene expression from RNA-seq experiment 1. Statistical analysis as in **Figure 2**. **p < 0.01, ****p < 0.0001, n.s. = non-significant.

**Figure 8:**
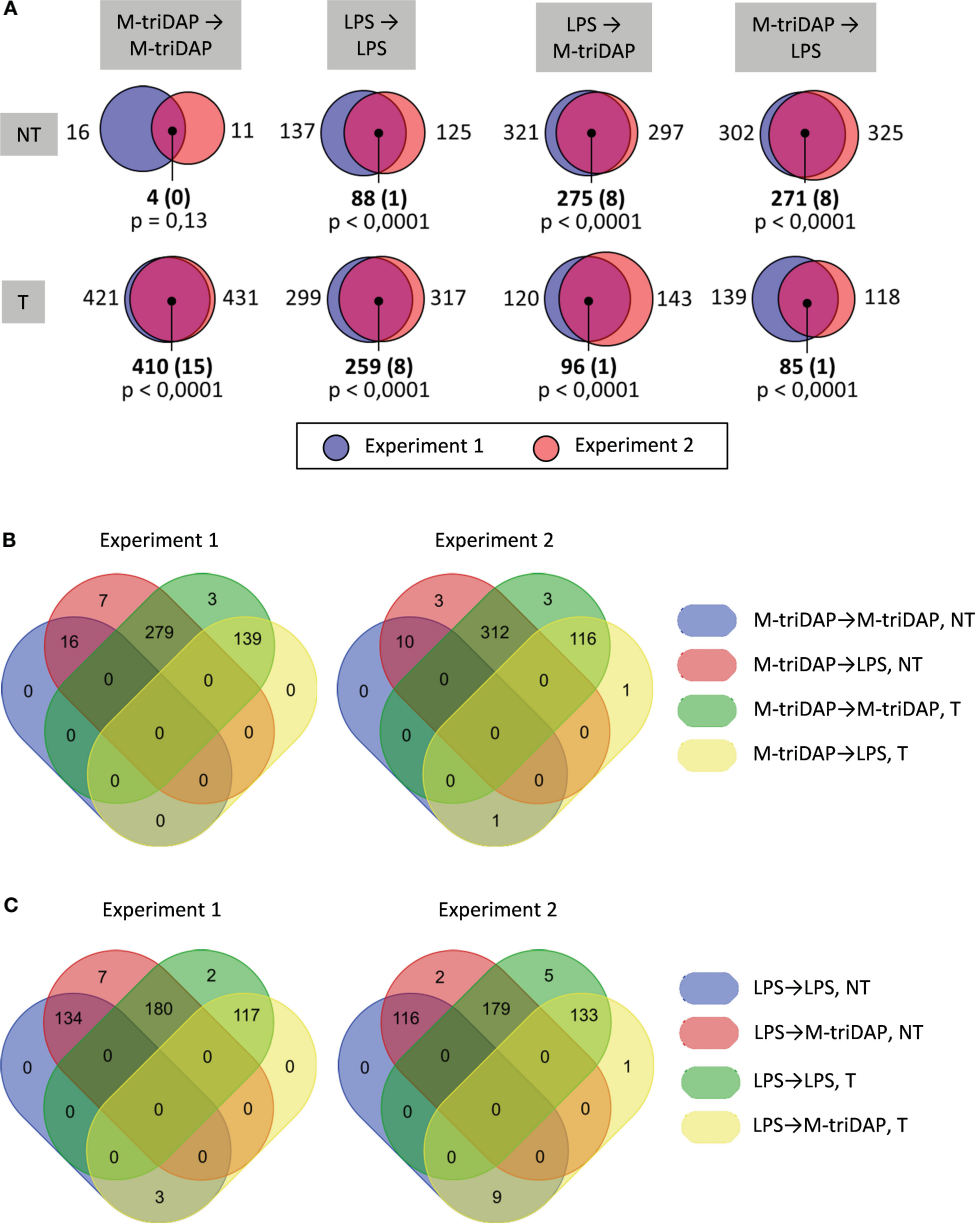
Intersections of T and NT gene sets identified in RNA-seq experiments. Only 446 genes inducible in naïve macrophages both by LPS and by M-triDAP in both RNA-seq experiments after 1 and/or 4 h of stimulation are included. **(A)** Sizes of T and NT gene sets identified in RNA-seq experiment 1 and 2 upon different stimulation sequences, and their intersections. **(B)** Venn diagrams showing intersections of T and NT gene sets upon M-triDAP➔M-triDAP and M-triDAP➔LPS stimulation. **(C)** Venn diagrams showing intersections of T and NT gene sets upon LPS➔LPS and LP➔M-triDAP stimulation.

When M-triDAP-tolerized macrophages were restimulated with LPS, the majority of analysed genes behaved as NT (**Figures 7A, B**), with good correspondence between two RNA-seq experiments (**Figure 8A**) and confirmatory RT-PCR experiments (Supplementary Figures S6A, B). About two-thirds of T genes observed in the M-triDAP➔M-triDAP conditions switched to NT in the M-triDAP➔LPS conditions (**Figure 8B**). Most of the NT genes identified in the M-triDAP➔LPS conditions were equally inducible by LPS in either naïve or M-triDAP-tolerized macrophages, which placed them in the NH category (**Figure 7**, **Supplementary Figures S6A, B**). NF-κB, p38 and JNK pathways in M-triDAP-tolerized cells were responsive to LPS stimulation (**Figure 6B**).

**Figure 9:**
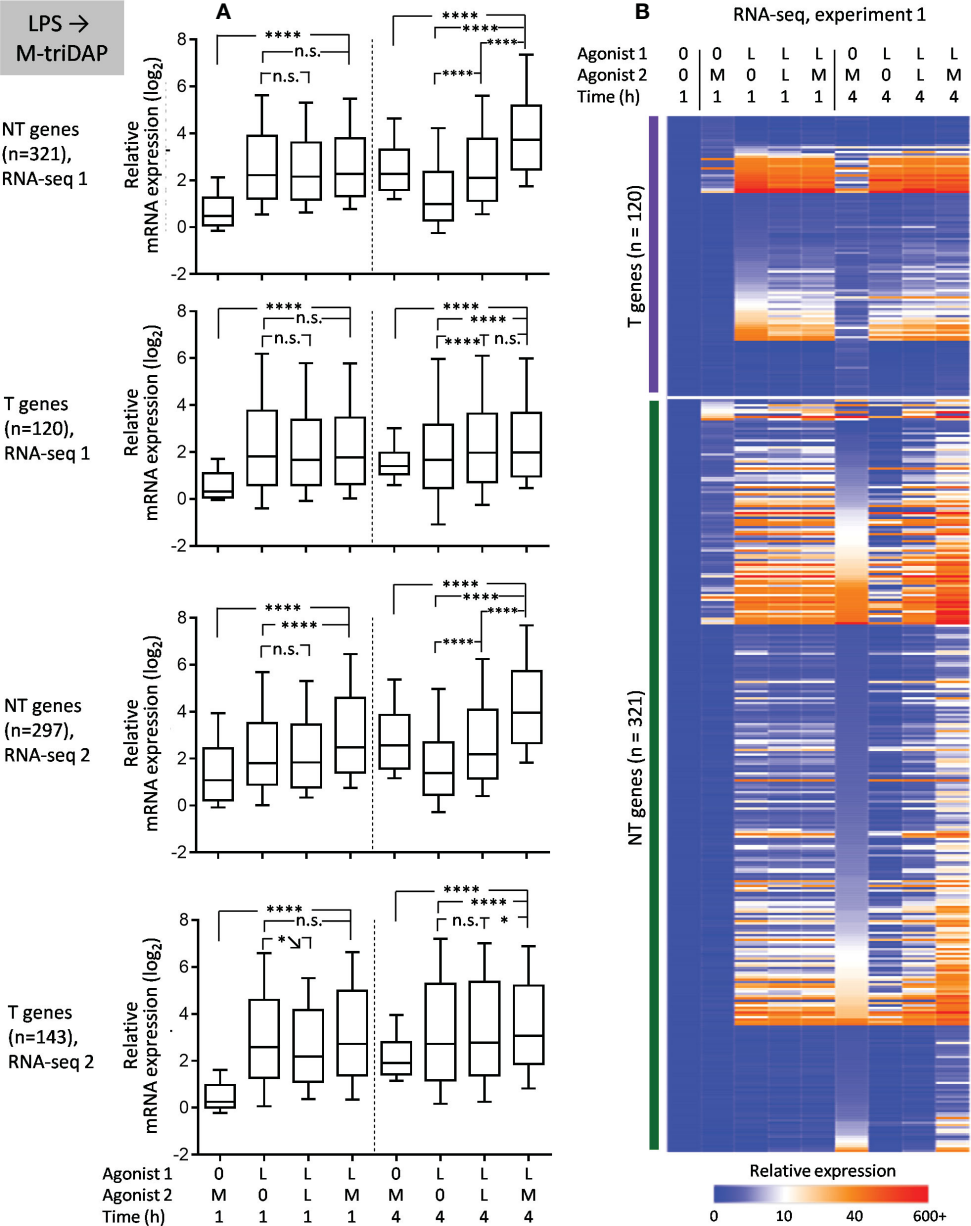
Responses of macrophages tolerized with LPS (L) to stimulation with M-triDAP (M). Only genes inducible in naïve macrophages both by M-triDAP and by LPS in both experiments after 1 and/or 4 h of stimulation are included (n = 446). **(A)** General statistics of T and NT gene expression in RNA-seq experiments 1 and 2 upon LPS➔M-triDAP stimulation. Responses of naïve macrophages to M-triDAP (0➔M-triDAP), of LPS-tolerized macrophages to LPS (LPS➔LPS) as well as residual LPS-induced gene expression (LPS➔0) are shown in parallel. **(B)** Heat map of relative gene expression from RNA-seq experiment 1. Statistical analysis as in **Figure 2**. *p < 0.05, ****p < 0.0001, n.s. = non-significant.

Upon LPS➔M-triDAP sequence, similarly, the majority of analysed genes showed an NT (NH) behavior (**Figures 9A, B**). Around half of genes classified as T and nearly all genes classified as NT in the LPS➔LPS settings fell in the NT category in the LPS➔M-triDAP settings (**Figure 8C**). A large proportion of NT genes were hyperinducible, i.e. M-triDAP induced their expression in LPS-tolerized cells to a greater extent than in naïve cells, as evident at 4 h of M-triDAP treatment (**Figure 8**, **Supplementary Figures S7A, B**). An enhanced response of LPS-tolerized macrophages to M-triDAP could be due to increased expression of *RIPK2* mRNA and corresponding RIP2 adaptor protein (**Supplementary Figures S7A, C**). In line with this, phosphorylation of p38 and JNK was strongly augmented in LPS-tolerized cells restimulated with M-triDAP for 60 min (**Figure 6B**). NF-κB pathway in LPS-tolerized macrophages was also responsive to M-triDAP stimulation (**Figure 6B**).

When presenting NT gene expression in bulk (**Figures 7A**, **9A**), responses to 2^nd^ stimulation, especially at 1 h, may be somewhat masked by genes having high residual expression after 1^st^ stimulation. However, when specifically examining genes with high inducibility at 1 h and low residual expression after 1^st^ stimulation, such as *TNF* in the LPS➔M-triDAP or M-triDAP➔LPS conditions or *IL6* in the M-triDAP➔LPS conditions (**Supplementary Figures S6A, S7A**), it is clear that their responses to 2^nd^ stimulation at either 1 or 4h are not significantly altered by the 1^st^ stimulus.

Altogether, a majority of genes behaving as T in homologous stimulation sequences, switch to NH upon cross-stimulation. These data contradict the idea of stable transcriptional silencing as the main mechanism of tolerance.

### Comparisons of NT and T genes characteristics

**Figure 10:**
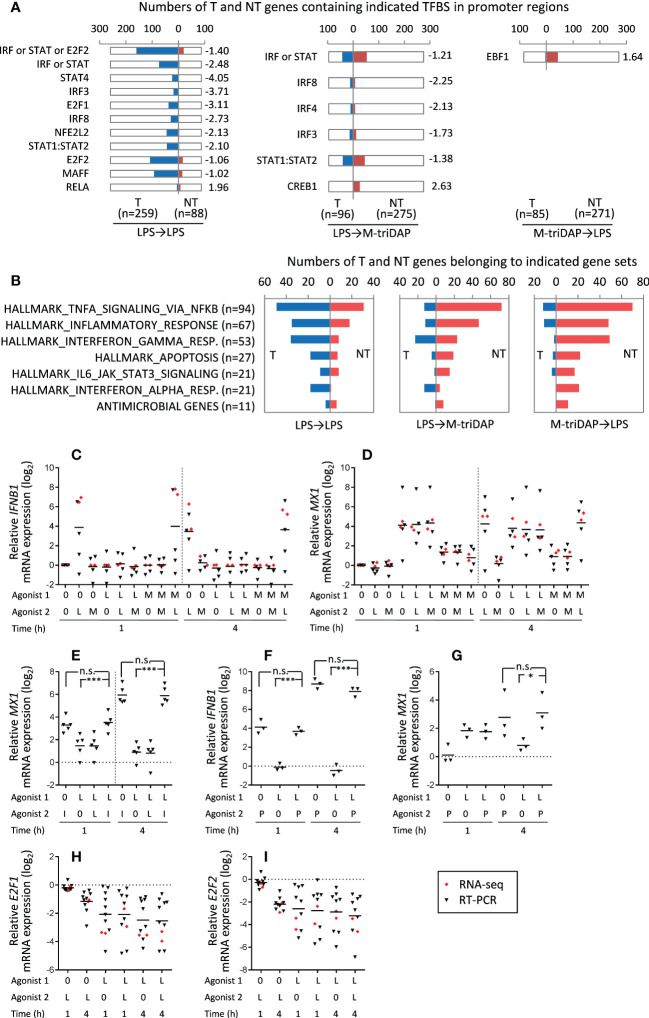
Regulation of T and NT gene expression in naïve and LPS-tolerized macrophages. **(A)** Numbers of T and NT genes containing indicated TFBS in their promoters. Only genes showing identical behavior in two RNA-seq experiments (see **Figure 8A**) are included in the analysis. Shown TFs satisfy following criteria: (i) TFBS are present in at least 10% of T or NT genes; (ii) enrichment score (ES) > 1; (iii) significant difference between frequencies of TFBS in T and NT gene sets (p < 0.01 by χ^2^-test). Numbers to the right of the plots indicate ES. **(B)** Representation of genes from indicated gene sets among T and NT genes upon different stimulation sequences. **(C, D)** Relative expression of *IFNB1* and *MX1* mRNA, respectively, after different stimulation sequences at 1 and 4 h of 2^nd^ stimulation. Horizontal bars denote means. **(E)** Effect of exogenous IFN-β **(I)** on *MX1* mRNA expression in naïve and LPS-tolerized macrophages. Cells were cultured for 24 h with medium (0) or with LPS (L, 100 ng/ml), then recultured for 1 or 4 h with medium, LPS (100 ng/ml) or rhIFN-β1b (10 U/ml). **(F, G)** Effect of liposomal poly-I:C (P) on *IFNB1* and *MX1* mRNA expression, respectively, in naïve and LPS-tolerized macrophages. Naïve or LPS-tolerized macrophages were recultured for 1 or 4 h with medium (0) or liposomal poly-I:C (1 μg/ml). **(H, I)** Expression of *E2F1* and *E2F2* mRNA in naïve and LPS-tolerized macrophages recultured with medium (0) or LPS (L) for 1 or 4 h. In **(C, D, H, I)**, red and black symbols denote RNA-seq and RT-PCR data, respectively. *p < 0.05, ***p < 0.001, n.s. = non-significant.

Since significant groups of genes behaved as T even upon cross-stimulation (**Figure 8**), we compared regulatory requirements of T and NT genes identified in different stimulation sequences. TFBS prediction analysis showed that in LPS➔M-triDAP and LPS➔LPS conditions, promoters of T genes were enriched with sites for TFs involved in induction of, or response to, type I IFN (IRF family, STAT1:STAT2, STAT4) (**Figure 10A**). Accordingly, members of the HALLMARK_INTERFERON_ALPHA_RESPONSE and to a lesser extent HALLMARK_INTERFERON_GAMMA_RESPONSE gene sets were preferentially classified as T in the LPS➔LPS and LPS➔M-triDAP conditions, but as NT in the M-triDAP➔LPS conditions (**Figure 10B**). It should be noted that the 446 genes selected for this analysis had to be inducible by M-triDAP, which excluded many IFN-regulated genes because they were induced only by LPS. When analysing all LPS-inducible genes in LPS➔LPS conditions, 35 members of HALLMARK_INTERFERON_ALPHA_RESPONSE gene set were present among T genes versus only one among NT (p < 0.001 by χ^2^-test).

It is known that IFN-β, acting auto- or paracrinely, mediates a large portion of the LPS-induced transcriptional program (38). Some of the IFN-regulated genes are also induced by M-triDAP, probably *via* IFN-independent mechanisms. Both RNA-seq and RT-PCR showed that *IFNB1* gene was induced by LPS in naïve or M-triDAP-tolerized macrophages, but not by LPS in LPS-tolerized macrophages or by M-triDAP in naïve or LPS-tolerized macrophages (**Figure 10C**). Accordingly, a key IFN response gene, *MX1*, was induced after 4 h of incubation with LPS but not with M-triDAP (**Figure 10D**). *MX1* behaved as T in LPS➔LPS conditions (**Figure 10D**, **Supplementary Figure S4**). However, exogenous IFN-β augmented *MX1* mRNA expression equally well in either naïve or LPS-tolerized macrophages (**Figure 10E**), indicating that ‘tolerization’ of *MX1* is due to lack of endogenous IFN-β, not to gene silencing. Since *IFNB1* gene is not induced by either LPS or M-triDAP in LPS-tolerized macrophages, lack of IFN-β in these conditions results in apparent ‘tolerization’ of LPS-inducible, IFN-regulated genes.

To check whether *IFNB1* gene itself is silenced in LPS-tolerized macrophages, we restimulated them with lipo-poly-I:C, liposome-encapsulated double-stranded RNA which activates *IFNB1* expression *via* RIG-like receptors (RLR), i.e. through a pathway distinct from those used by TLRs or NODs (39). As shown in **Figure 10F**, *IFNB1* gene remained fully responsive to lipo-poly-I:C in LPS-tolerized cells, and comparable *MX1* induction was observed at 4 h of lipo-poly-I:C stimulation in either LPS-tolerized or naïve macrophages (**Figure 10G**). Thus, lack of *IFNB1* expression in LPS-tolerized macrophages is probably due to inhibition of TRIF-dependent signaling pathway downstream of TLR4. Together, these data point at the deficit of endogenous IFN-β as an important factor contributing to LPS tolerance.

In addition, T gene promoters in the LPS➔LPS conditions were enriched with E2F1 and E2F2 binding sites (E2F1 target genes being a subset of E2F2 target genes) (**Figure 10A**). Different studies report on both positive and negative roles of E2F1 and E2F2 transcription factors in responses to pro-inflammatory stimuli (40–43). Interestingly, levels of *E2F1* and *E2F2* mRNAs were strongly downregulated in LPS-tolerized cells as compared to naïve macrophages (**Figures 10H, I**). These data allow a hypothesis that deficit of E2F1 and E2F2 may represent an additional mechanism of LPS tolerance. Overall, in LPS➔LPS conditions, 160 out of 259 T genes (62%) contained IRF3/8, STAT1:STAT2, STAT4 or E2F2 binding targets *versus* only 20 such genes out of 88 (23%) in the NT class (p < 0.0001 by χ^2^ test).

We cannot draw conclusions about remaining T genes in LPS➔LPS and LPS➔M-triDAP conditions. These genes may be subject to additional regulatory mechanisms, such as post-transcriptional silencing by micro-RNA. The same may apply to the M-triDAP➔LPS sequence, where no TFBS significantly associated with T genes were found (**Figure 10A**).

## Discussion

Tolerance is a fundamental mechanism of innate immunity which serves to limit inflammatory responses, but may also underlie immune paralysis observed in sepsis patients. Specific mechanisms of innate tolerance are incompletely understood. Epigenetic and signaling-based mechanisms have been proposed (5, 12), but neither of them adequately explains all aspects of tolerance. Epigenetic scenario predicts that cells treated by an agonist of one PRR would become unresponsive to agonists of other PRRs as well (cross-tolerized). Signaling-based scenarios imply that responses induced through PRRs signaling *via* distinct pathways should not be cross-tolerized. Here, we tolerized human macrophages with agonists of TLR4 or NOD1 receptors, which utilize distinct signaling pathways, and analysed responses of tolerized cells to homologous restimulation and cross-stimulation. Whenever needed, we also used stimuli signaling through additional receptors and pathways, such as type I IFN receptors and RLRs.

A stereotype response of macrophages to PRR stimulation is a rapid increase of glycolytic rate, which is reflected by the rise of ECAR within 1 h of stimulation (34, 37). This response has been explained by PI3K-Akt-dependent intracellular redistribution of hexokinase-II and GLUT1 glucose transporter (44–46) and does not require gene expression *de novo* (37). ECAR elevation was similarly blocked in either LPS- or M-triDAP-tolerized macrophages restimulated with the same agonist (**Figures 2A**, **5A**), indicating inhibition of PI3K-Akt-dependent signaling pathways downstream of respective receptors. Similarly, p38 and JNK pathways were not activated in either LPS- or M-triDAP-tolerized macrophages in response to homologous restimulation (**Figure 6B**). Interestingly, the NF-κB pathway responded to homologous restimulation in LPS-tolerized but not in M-triDAP-tolerized macrophages. This shows that NOD1-dependent signaling in M-triDAP-tolerized macrophages is suppressed more profoundly than TLR4-dependent signaling in LPS-tolerized cells. At the same time, in cross-stimulation conditions, activation of NF-κB and MAP kinases was not inhibited compared to naïve macrophages, indicating that signal transduction in both LPS- and M-triDAP-tolerized macrophages is blocked at receptor-proximal stages, as suggested earlier (3, 5).

To analyse behavior of genes in tolerized macrophages, we compared expression of each gene in tolerized macrophages upon restimulation (agonist 1 ➔ agonist 2) not only with the expression in naïve macrophages (0 ➔ agonist 2), but also with the expression in tolerized cells recultured without agonists (agonist 1 ➔ 0), which allowed to distinguish responses to secondary stimulation from continuing responses to primary stimulation. This was unlike some earlier works, where genes were classified into T and NT based on simple ratios of expression after 1^st^ and 2^nd^ stimulation with an agonist, without taking into account residual expression after the 1^st^, tolerizing stimulation (4, 12, 47); as a consequence, the number of genes with increased responses to secondary stimulation in those works may have been overestimated. Second, studying two time points (1 and 4 h after primary/secondary stimulation) allowed us to roughly assess and compare kinetics of gene expression in naïve and tolerized macrophages.

Transcriptional responses of M-triDAP- and LPS-tolerized macrophages to homologous restimulation were somewhat different. M-triDAP-tolerized macrophages showed virtually no response (**Figure 5**, **Supplementary Figure S5**), whereas in LPS-tolerized macrophages, a subset of LPS-inducible genes retained certain responsiveness to LPS, which is in agreement with the incomplete blockade of TLR4-dependent signaling in LPS-tolerized cells. By analysing kinetics of gene expression, we found that many of these NT genes in LPS-tolerized cells follow a pattern of sustained expression (SE), whereby a gene has an elevated residual expression after the 24-hour tolerizing LPS treatment, which drops after removal of LPS from the medium, but is maintained at an increased level when LPS is added again for the 2^nd^ stimulation. However, we did not find genes that would consistently respond to LPS restimulation equally or stronger than to primary stimulation (taking into account residual gene expression after primary LPS stimulation). Existence of genes with increased responsiveness to homologous restimulation is difficult to explain by signaling-based mechanisms. Virtual absence of such genes in M-triDAP- or LPS-tolerized macrophages eliminates an important drawback of the signaling-based scenario of tolerance. At the same time, it should be noted that transcriptomes of tolerized macrophages are significantly altered as the result of primary stimulation (**Supplementary Figure S2**), and expression of many genes in tolerized macrophages is higher than at 1 or 4 h of primary stimulation (e.g., *PTGES* and *FPR1* in LPS➔LPS conditions), which can be explained by high mRNA stability and/or continuing transcription (**Figure 4**). However, most of such genes do not change their mRNA expression upon homologous restimulation and are therefore classified as T.

Consequently, we did not observe a dichotomy between T/pro-inflammatory and NT/antimicrobial genes reported in LPS-tolerized murine macrophages (12). As noted above, our definition of NH genes aims to capture genes with the same characteristics as NT genes in the study by Foster et al. (12), but takes into account residual gene expression. When we analysed the 196 genes defined as NT by Foster et al. (12), only 126 were significantly expressed in our dataset, only 56 were induced by LPS at any point, only 4 were NT as defined by our criteria, and none was NH. Moreover, many of the NT genes defined by our criteria (i.e., showing any response to LPS restimulation) encode pro-inflammatory proteins (**Figure 3B**, **Supplementary Figure S3**). It is possible that these discrepancies are due to inter-species differences (murine macrophages in (12) vs. human ones in our study).

Promoters of T genes in LPS-tolerized macrophages carry inhibitory covalent modifications of histones and lack activating histone modifications upon LPS rechallenge (12, 13, 48, 49). These epigenetic marks are proposed to underlie T gene silencing; however, they may also be explained by lack of activating inputs due to blockade of signaling pathways downstream of TLR4. We took advantage of cross-stimulating macrophages with agonists of TLR4 and NOD1 receptors, which signal through distinct pathways. As already noted, M-triDAP- and LPS-tolerized macrophages retain the ability to activate distal portions of signaling pathways that are common to both receptors (NF-κB, MAP kinases). Consequently, the majority of genes not responding to homologous restimulation showed either normal or increased responses to cross-stimulation, as compared to responses in naïve macrophages (**Figures 7**-**9**). Obviously, the inhibitory epigenetic marks observed in cells tolerized to a particular PRR agonist can easily be overcome by stimulation of another PRR which signals through a distinct pathway that remains active. It follows that inhibition of signaling pathways downstream of specific PRRs, rather than epigenetic silencing, plays the key role in innate tolerance. Our data are in line with earlier studies which showed that pairs of PRRs signaling through distinct proximal adapters do not cross-tolerize (5, 49). At the same time, we should point out that we used a relatively short-term tolerization (24 h), which is most pertinent to early stages of sepsis or to fulminant sepsis. Phenotypic alterations induced by prolonged PRR agonist treatment, such as innate immune memory, trained immunity or exhaustion, may be more dependent on epigenetic mechanisms than short-term tolerance (15, 50).

In search for additional factors contributing to tolerance, we found that many T genes in LPS➔LPS and LPS➔M-triDAP conditions are IFN-regulated. *IFNB1* gene itself behaves as T in LPS-tolerized cells restimulated with LPS, and it is not inducible by M-triDAP. However, *IFNB1* gene is not silenced in LPS-tolerized macrophages, because it remains fully responsive to an RLR agonist (**Figure 10F**). In addition, IFN-regulated genes such as *MX1*, which behave as T in LPS-tolerized cells, remain fully responsive to exogenous IFN-β (**Figure 10E**). This means that ‘pseudo-tolerization’ of IFN-regulated genes in LPS-tolerized cells is due to deficit of endogenous IFN-β, which is itself caused by blockade of TRIF-dependent signaling pathway downstream of TLR4 (or by inability of M-triDAP to induce *IFNB1* expression) rather than by silencing of *IFNB1* gene. In view of our data, recombinant IFN-β or RLR agonists might be used in the immunoparalysis stage of bacterial sepsis in order to restore protective gene expression.

Another mechanism that could potentially contribute to LPS tolerance may be deficit of E2F1 and/or E2F2 transcription factors. In LPS-tolerized macrophages, *E2F1* and *E2F2* mRNA expression is strongly downregulated, while promoters containing E2F1 and/or E2F2 binding sites are enriched among T genes (**Figures 10A, H, I**). Downregulation of *E2F1* and *E2F2* expression may be mediated by auto/paracrine effects of pro-inflammatory cytokines such as TNF (51). E2F family transcription factors are critical regulators of the cell cycle (52), whereas their involvement in innate immune responses is less well established, with both pro- and anti-inflammatory roles reported (40–43). As examples of pro-inflammatory effects, physical and functional interaction between E2F1 and RelA has been demonstrated (43, 53), and knock-down or knock-out of endogenous *E2F1* results in decreased LPS-induced pro-inflammatory cytokine production *in vitro* and *in vivo* (39, 51). E2F2 may act as a positive regulator of TLR4-dependent signaling (54). Thus, deficit of E2F1 and E2F2 may impair expression of a subset of LPS-inducible, E2F-dependent genes in LPS-tolerized macrophages, although this hypothesis requires further experimental testing. Interestingly, a decrease of *E2F1* and *E2F2* mRNA is detected in M-triDAP-tolerized macrophages as well (data not shown), yet it has no obvious consequences because NOD1-dependent signal transduction in M-triDAP-tolerized cells is blocked at a more upstream, receptor-proximal stage (see above).

In all, we show that the behavior of genes in tolerized macrophages is not predetermined, but depends on specific sequences of the first (tolerizing) and the second (resolving) stimulus. Our data are consistent with the idea that innate tolerance can be adequately explained by signaling-based mechanisms. Signaling pathways that remain uninhibited can be used to restore responsiveness of tolerized cells.

## Data availability statement

The data presented in the study are deposited in the GEO repository, accession number GSE207510, https://www.ncbi.nlm.nih.gov/geo/query/acc.cgi?acc=GSE207510.

## Ethics statement

The studies involving human participants were reviewed and approved by Ethical Committee of the Institute of Immunology. The patients/participants provided their written informed consent to participate in this study.

## Author contributions

AM conducted experiments and analysed results, PM conducted Seahorse metabolic flux assays, GC isolated blood mononuclear cells, MP designed and conducted experiments, analysed results and wrote the paper. All authors approved the final version.
