# Supplementary material for: Inhibition of specific signaling pathways rather than epigenetic silencing of effector genes is the leading mechanism of innate tolerance
Source: Front Immunol. 2023 Jan 26;14:1006002. doi: 10.3389/fimmu.2023.1006002 (PMC9909295; doi:10.3389/fimmu.2023.1006002)
Supplement: Supplementary file 1 [file Table_1.docx]

**Supplementary Table 1.** PCR primers used to assess mature mRNA expression.

| Gene symbol | Gene full name | Primer | Sequence (5’ 🡪 3’) |
| --- | --- | --- | --- |
| *E2F1* | E2F Transcription Factor 1 | Forward | GAAGCGGCGCATCTATGACA |
|  |  | Reverse | GTCCTGGGTCAACCCCTCAA |
| *E2F2* | E2F Transcription Factor 2 | Forward | GGGGAATGTTTGAAGACCCCAC |
|  |  | Reverse | AAAGTTGCCAACAGCACGGA |
| *FPR1* | Formyl Peptide Receptor 1 | Forward | CTACCCAGAGCAAGACCACAG |
|  |  | Reverse | GTCCCTCCAGAGATGTTCGTG |
| *GAPDH* | Glyceraldehyde 3-phosphate dehydrogenase | Forward | CAGCCTCCCGCTTCGCTCTC |
|  |  | Reverse | ACCAGGCGCCCAATACGACC |
| *IFNB1* | Interferon Beta 1 | Forward | AGCAGTCTGCACCTGAAAAG |
|  |  | Reverse | CTGACTATGGTCCAGGCACAG |
| *IL1B* | Interleukin 1B | Forward | GAGCTCGCCAGTGAAATGATG |
|  |  | Reverse | TGGTGGTCGGAGATTCGTAG |
| *IL6* | Interleukin 6 | Forward | GCCACTCACCTCTTCAGAACG |
|  |  | Reverse | TCAGCCATCTTTGGAAGGTTCA |
| *IL10* | Interleukin 10 | Forward | ACCAAGACCCAGACATCAAGG |
|  |  | Reverse | CCTTGCTCTTGTTTTCACAGGG |
| *IL12B* | Interleukin 12B | Forward | ATTCTGCGTTCAGGTCCAGG |
|  |  | Reverse | AGAACCTAACTGCAGGGCAC |
| *IL23A* | Interleukin 23 subunit alpha | Forward | GCAAAAAGATGCTGGGGAGC |
|  |  | Reverse | TCTCTTAGATCCATGTGTCCCAC |
| *MX1* | MX dynamin like GTPase 1 | Forward | GGATTTTGGGGCTTTCCAGTC |
|  |  | Reverse | CCGTACGTCTGGAGCATGAAG |
| *NFKB1*  *(p50)* | Nuclear factor kappa B subunit 1 | Forward | AATGGGCTACACCGAAGCAA |
|  |  | Reverse | TTGCGGAAGGATGTCTCCAC |
| *NFKBIA* | NFKB inhibitor alpha | Forward | AAGCAGCAGCTCACCGAG |
|  |  | Reverse | ACAGCCAAGTGGAGTGGAG |
| *NOD1* | nucleotide binding oligo-merization domain containing 1 | Forward | TCAGATCACAGCTAAGGGGAC |
|  |  | Reverse | TAGACTTTGGCCTCCTCTGG |
| *PIM2* | Pim-2 Proto-Oncogene, Serine/Threonine Kinase | Forward | GGTGGCCATCAAAGTGATTCC |
|  |  | Reverse | CACCTGCACCCACTTTCCATA |
| *PKIG* | CAMP-Dependent Protein Kinase Inhibitor Gamma | Forward | GAATGCGGTCCCTGACATCC |
|  |  | Reverse | TCAAGACGAGGTGGTCCCAT |
| *PTGES* | Prostaglandin E Synthase | Forward | CCCAGTATTGCAGGAGCGA |
|  |  | Reverse | CGACAAAAGGGTTAGGACCCA |
| *RELA*  *(p65)* | RELA proto-oncogene, NF-kB subunit (p65) | Forward | ACCGGATTGAGGAGAAACGTA |
|  |  | Reverse | GACGTAAAGGGATAGGGCTGG |
| *RIPK2*  *(RIP2)* | Receptor interacting serine/threonine kinase 2 | Forward | TTTGGGAATTTGCAATGAGCCTG |
|  |  | Reverse | GCGAAATCTCAATGGCCAAGC |
| *RIPOR2* | RHO Family Interacting Cell Polarization Regulator 2 | Forward | GCACCATTGAAGTGGAGCTAGA |
|  |  | Reverse | TTCATATTGATCTCCAGGACAGAGG |
| *TNF* | Tumor necrosis factor | Forward | TCGGCCCCCAGAGGGAAGAG |
|  |  | Reverse | CGGCGGTTCAGCCACTGGAG |
| *TNFAIP3*  *(A20)* | TNF alpha induced protein 3 | Forward | AGGTTCCAGAACACCATTCCG |
|  |  | Reverse | GTTCGAGGCACATCTCTGCG |
| *WNT5A* | Wnt Family Member 5A | Forward | CATCGACTATGGCTACCGCTT |
|  |  | Reverse | AGGCTACATGAGCCGGACA |
