# Supplementary material for: Inhibition of specific signaling pathways rather than epigenetic silencing of effector genes is the leading mechanism of innate tolerance
Source: Front Immunol. 2023 Jan 26;14:1006002. doi: 10.3389/fimmu.2023.1006002 (PMC9909295; doi:10.3389/fimmu.2023.1006002)
Supplement: Supplementary file 2 [file Table_2.docx]

**Supplementary Table 2.** PCR primers used to detect nascent transcripts.

| Gene symbol | Gene full name | Primer position | Primer | Sequence (5’ 🡪 3’) |
| --- | --- | --- | --- | --- |
| *RNU6 (U6)* | RNA, U6 Small Nuclear 1 | exon 1 | Forward | CTCGCTTCGGCAGCACA |
|  |  |  | Reverse | AACGCTTCACGAATTTGCGT |
| *FPR1* | Formyl Peptide Receptor 1 | exon 2 | Forward | ACAAAGGTGACTGCAAATACCAG |
|  |  |  | Reverse | TCCCCACGAACATCTCTGGA |
| *IL1B* | Interleukin 1B | exon 7 | Forward | CACTGCTACTTCTTGCCCCCT |
|  |  |  | Reverse | TGACAGAAACCACGGCCACA |
| *IL6* | Interleukin 6 | exon 5 | Forward | CCTGACCCAACCACAAATGC |
|  |  |  | Reverse | ACAACAATCTGAGGTGCCCAT |
| *PTGES* | Prostaglandin E Synthase | exon 3 | Forward | TGTAGGTCACGGAGCGGATG |
|  |  |  | Reverse | ACCGGAACGACATGGAGACC |
| *TNF* | Tumor necrosis factor | exon 4 | Forward | CCCATCTATCTGGGAGGGGT |
|  |  |  | Reverse | GCGTTTGGGAAGGTTGGATG |
| *WNT5A* | Wnt Family Member 5A | exon 5 | Forward | TGGCACCCACTACTTGCACA |
|  |  |  | Reverse | TCAAGACCGTGCAGACGGAG |
