# Supplementary material for: Inhibition of specific signaling pathways rather than epigenetic silencing of effector genes is the leading mechanism of innate tolerance
Source: Front Immunol. 2023 Jan 26;14:1006002. doi: 10.3389/fimmu.2023.1006002 (PMC9909295; doi:10.3389/fimmu.2023.1006002)
Supplement: Supplementary file 3 [file Presentation_1.pptx]

## Slide 1
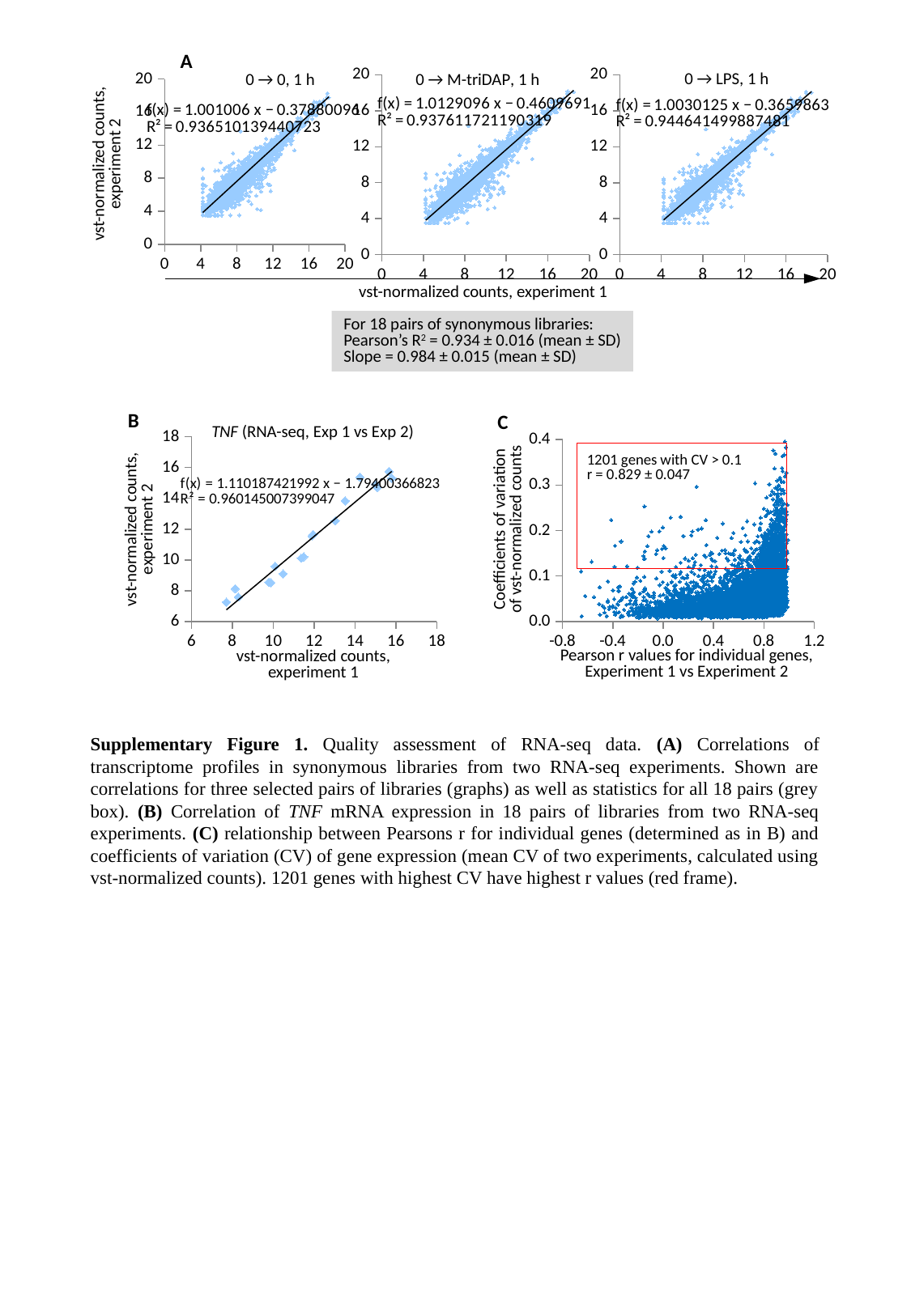

A
### Chart
| Category | |
|---|---|0 → LPS, 1 h
0 → 0, 1 h
### Chart
| Category | |
|---|---|
### Chart
| Category | |
|---|---|0 → M-triDAP, 1 h
vst-normalized counts,
experiment 2
vst-normalized counts, experiment 1
For 18 pairs of synonymous libraries:
Pearson’s R2 = 0.934 ± 0.016 (mean ± SD)
Slope = 0.984 ± 0.015 (mean ± SD)
B
C
TNF (RNA-seq, Exp 1 vs Exp 2)
### Chart
| Category | |
|---|---|
### Chart
| Category | |
|---|---|
1201 genes with CV > 0.1
r = 0.829 ± 0.047
vst-normalized counts,
experiment 2
Coefficients of variation
of vst-normalized counts
Pearson r values for individual genes,
Experiment 1 vs Experiment 2
vst-normalized counts,
experiment 1
Supplementary Figure 1. Quality assessment of RNA-seq data. (A) Correlations of transcriptome profiles in synonymous libraries from two RNA-seq experiments. Shown are correlations for three selected pairs of libraries (graphs) as well as statistics for all 18 pairs (grey box). (B) Correlation of TNF mRNA expression in 18 pairs of libraries from two RNA-seq experiments. (C) relationship between Pearsons r for individual genes (determined as in B) and coefficients of variation (CV) of gene expression (mean CV of two experiments, calculated using vst-normalized counts). 1201 genes with highest CV have highest r values (red frame).
1

## Slide 2
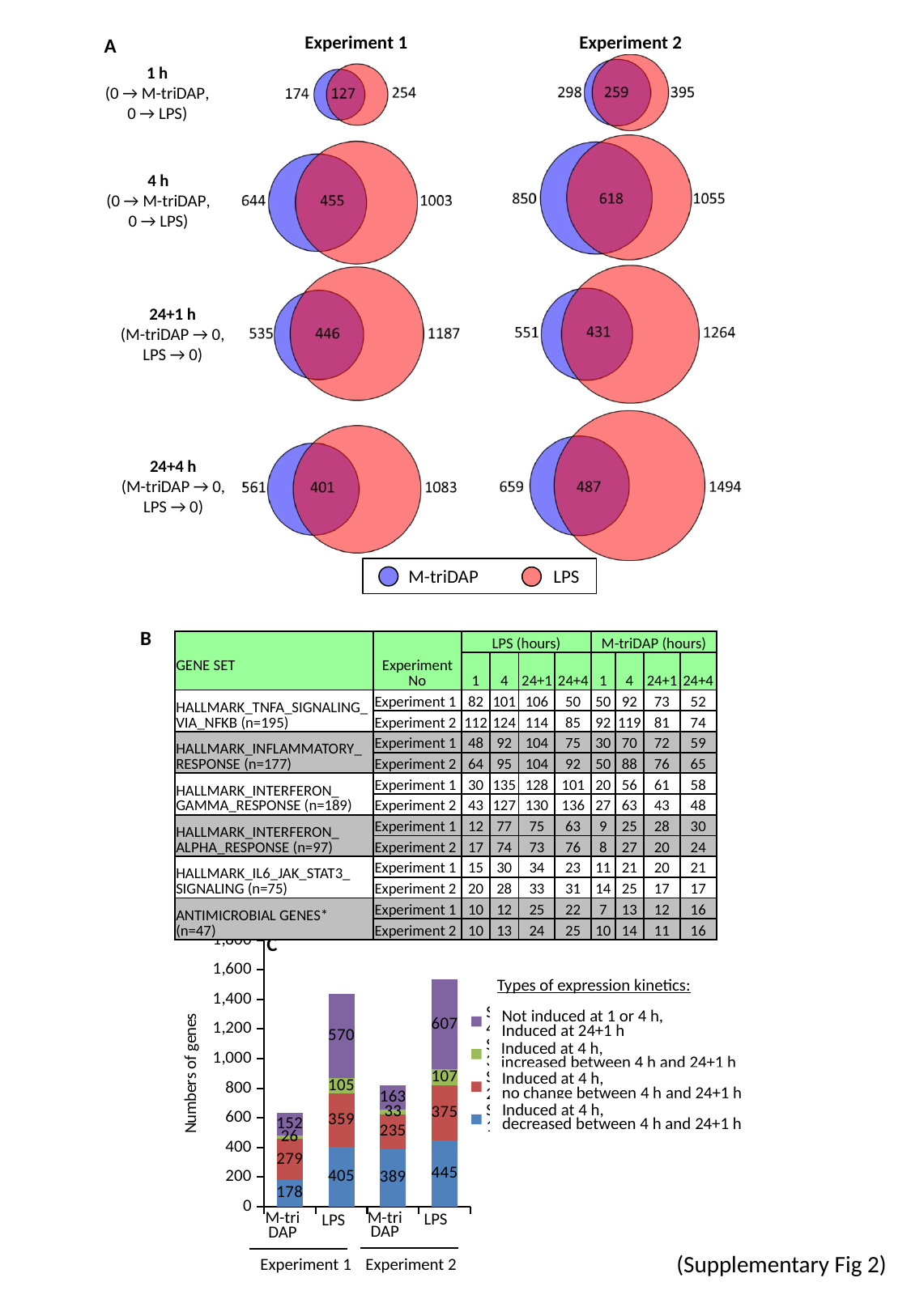

Experiment 1
Experiment 2
A
1 h
(0 → M-triDAP,
0 → LPS)
4 h
(0 → M-triDAP,
0 → LPS)
24+1 h
(M-triDAP → 0,
LPS → 0)
24+4 h
(M-triDAP → 0,
LPS → 0)
LPS
M-triDAP
B
| GENE SET | Experiment No | LPS (hours) | | | | M-triDAP (hours) | | | |
| --- | --- | --- | --- | --- | --- | --- | --- | --- | --- |
| | | 1 | 4 | 24+1 | 24+4 | 1 | 4 | 24+1 | 24+4 |
| HALLMARK\_TNFA\_SIGNALING\_ VIA\_NFKB (n=195) | Experiment 1 | 82 | 101 | 106 | 50 | 50 | 92 | 73 | 52 |
| | Experiment 2 | 112 | 124 | 114 | 85 | 92 | 119 | 81 | 74 |
| HALLMARK\_INFLAMMATORY\_ RESPONSE (n=177) | Experiment 1 | 48 | 92 | 104 | 75 | 30 | 70 | 72 | 59 |
| | Experiment 2 | 64 | 95 | 104 | 92 | 50 | 88 | 76 | 65 |
| HALLMARK\_INTERFERON\_ GAMMA\_RESPONSE (n=189) | Experiment 1 | 30 | 135 | 128 | 101 | 20 | 56 | 61 | 58 |
| | Experiment 2 | 43 | 127 | 130 | 136 | 27 | 63 | 43 | 48 |
| HALLMARK\_INTERFERON\_ ALPHA\_RESPONSE (n=97) | Experiment 1 | 12 | 77 | 75 | 63 | 9 | 25 | 28 | 30 |
| | Experiment 2 | 17 | 74 | 73 | 76 | 8 | 27 | 20 | 24 |
| HALLMARK\_IL6\_JAK\_STAT3\_ SIGNALING (n=75) | Experiment 1 | 15 | 30 | 34 | 23 | 11 | 21 | 20 | 21 |
| | Experiment 2 | 20 | 28 | 33 | 31 | 14 | 25 | 17 | 17 |
| ANTIMICROBIAL GENES\* (n=47) | Experiment 1 | 10 | 12 | 25 | 22 | 7 | 13 | 12 | 16 |
| | Experiment 2 | 10 | 13 | 24 | 25 | 10 | 14 | 11 | 16 |
C
### Chart
| Category | | | | |
|---|---|---|---|---|Types of expression kinetics:
Not induced at 1 or 4 h,
Induced at 24+1 h
Induced at 4 h,
increased between 4 h and 24+1 h
Induced at 4 h,
no change between 4 h and 24+1 h
Induced at 4 h,
decreased between 4 h and 24+1 h
LPS
LPS
M-tri
DAP
M-tri
DAP
(Supplementary Fig 2)
Experiment 2
Experiment 1

## Slide 3
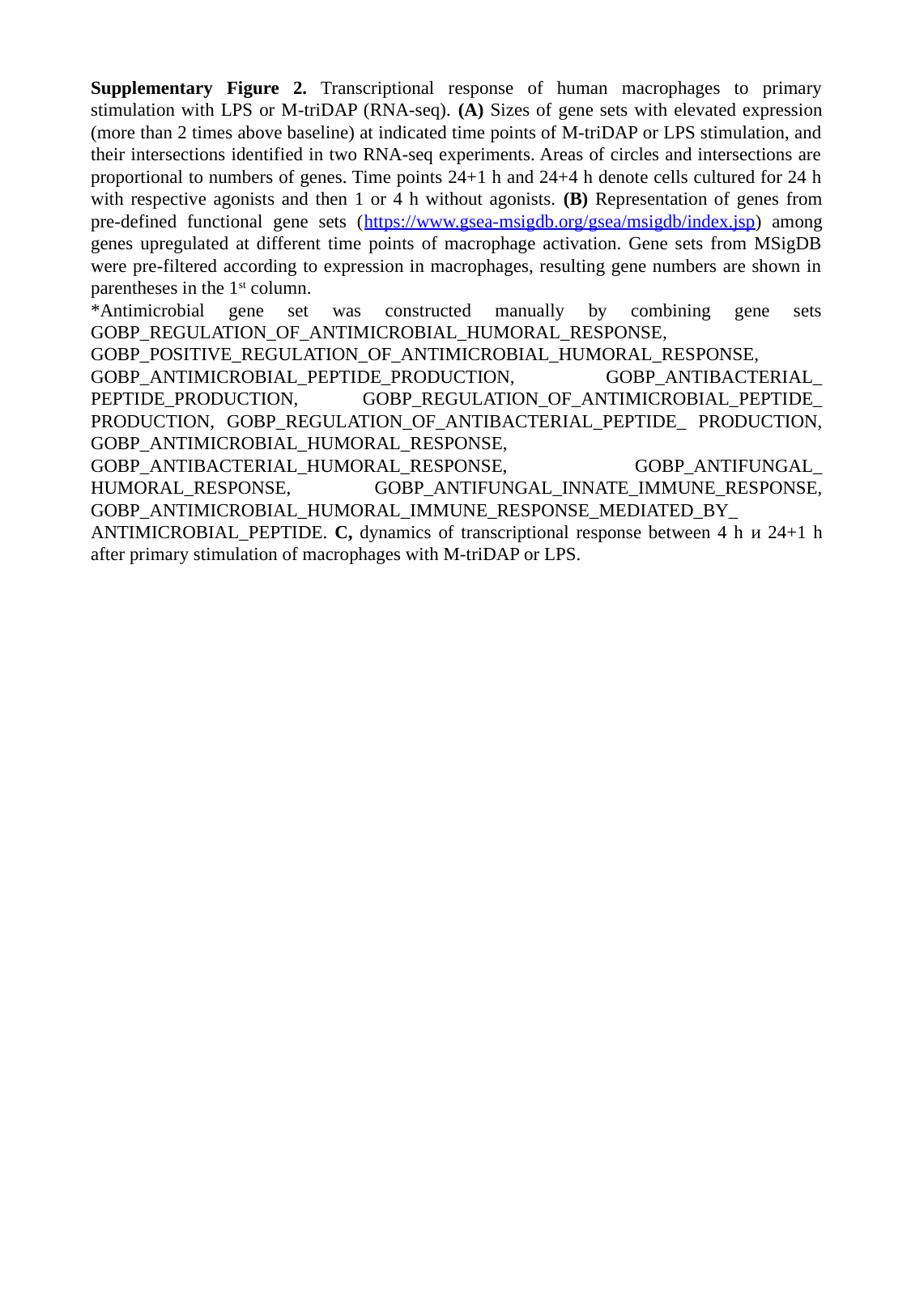

Supplementary Figure 2. Transcriptional response of human macrophages to primary stimulation with LPS or M-triDAP (RNA-seq). (A) Sizes of gene sets with elevated expression (more than 2 times above baseline) at indicated time points of M-triDAP or LPS stimulation, and their intersections identified in two RNA-seq experiments. Areas of circles and intersections are proportional to numbers of genes. Time points 24+1 h and 24+4 h denote cells cultured for 24 h with respective agonists and then 1 or 4 h without agonists. (B) Representation of genes from pre-defined functional gene sets (https://www.gsea-msigdb.org/gsea/msigdb/index.jsp) among genes upregulated at different time points of macrophage activation. Gene sets from MSigDB were pre-filtered according to expression in macrophages, resulting gene numbers are shown in parentheses in the 1st column.
*Antimicrobial gene set was constructed manually by combining gene sets GOBP_REGULATION_OF_ANTIMICROBIAL_HUMORAL_RESPONSE, GOBP_POSITIVE_REGULATION_OF_ANTIMICROBIAL_HUMORAL_RESPONSE, GOBP_ANTIMICROBIAL_PEPTIDE_PRODUCTION, GOBP_ANTIBACTERIAL_ PEPTIDE_PRODUCTION, GOBP_REGULATION_OF_ANTIMICROBIAL_PEPTIDE_ PRODUCTION, GOBP_REGULATION_OF_ANTIBACTERIAL_PEPTIDE_ PRODUCTION, GOBP_ANTIMICROBIAL_HUMORAL_RESPONSE, GOBP_ANTIBACTERIAL_HUMORAL_RESPONSE, GOBP_ANTIFUNGAL_ HUMORAL_RESPONSE, GOBP_ANTIFUNGAL_INNATE_IMMUNE_RESPONSE, GOBP_ANTIMICROBIAL_HUMORAL_IMMUNE_RESPONSE_MEDIATED_BY_ ANTIMICROBIAL_PEPTIDE. C, dynamics of transcriptional response between 4 h и 24+1 h after primary stimulation of macrophages with M-triDAP or LPS.

## Slide 4
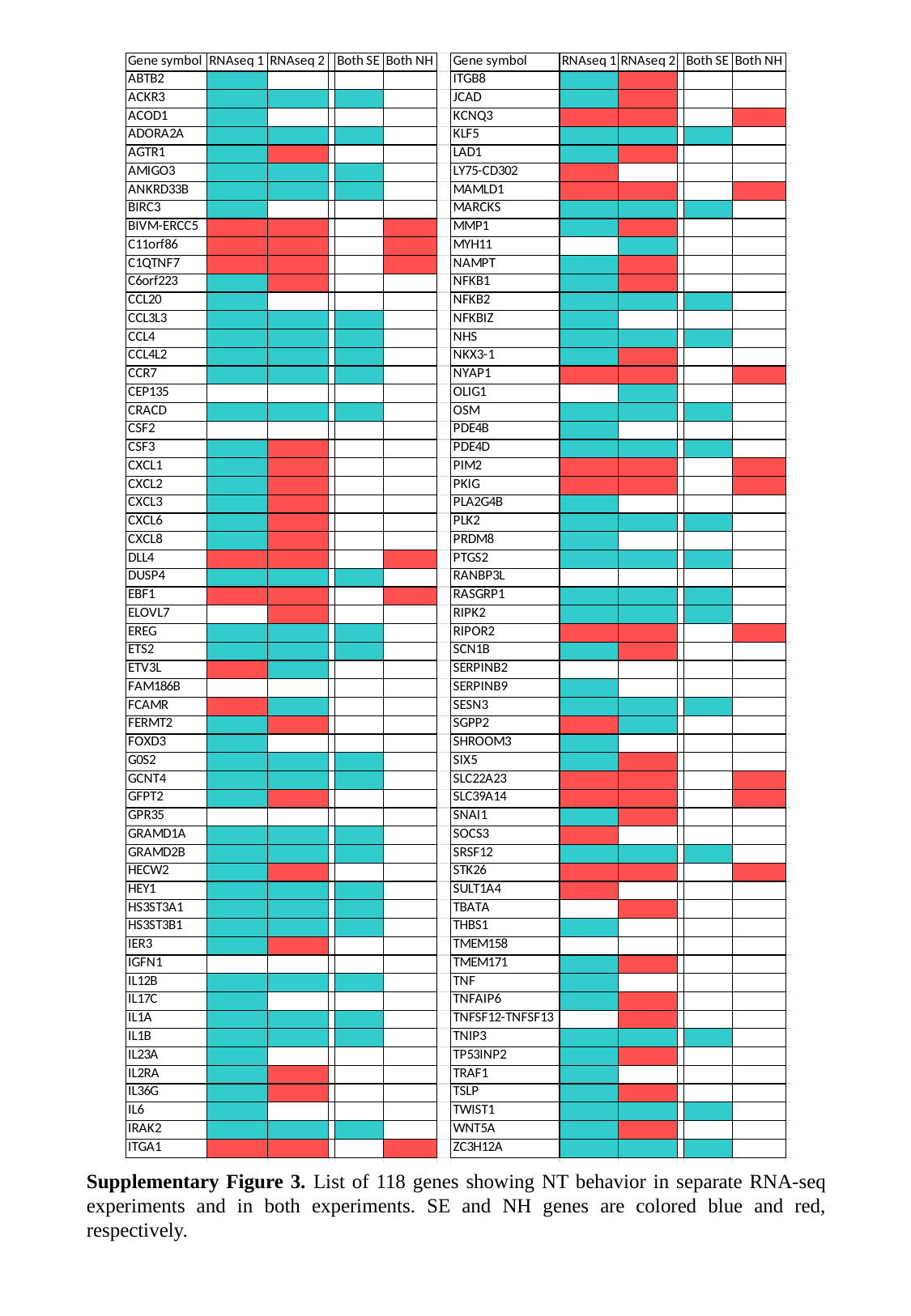

Supplementary Figure 3. List of 118 genes showing NT behavior in separate RNA-seq experiments and in both experiments. SE and NH genes are colored blue and red, respectively.

## Slide 5
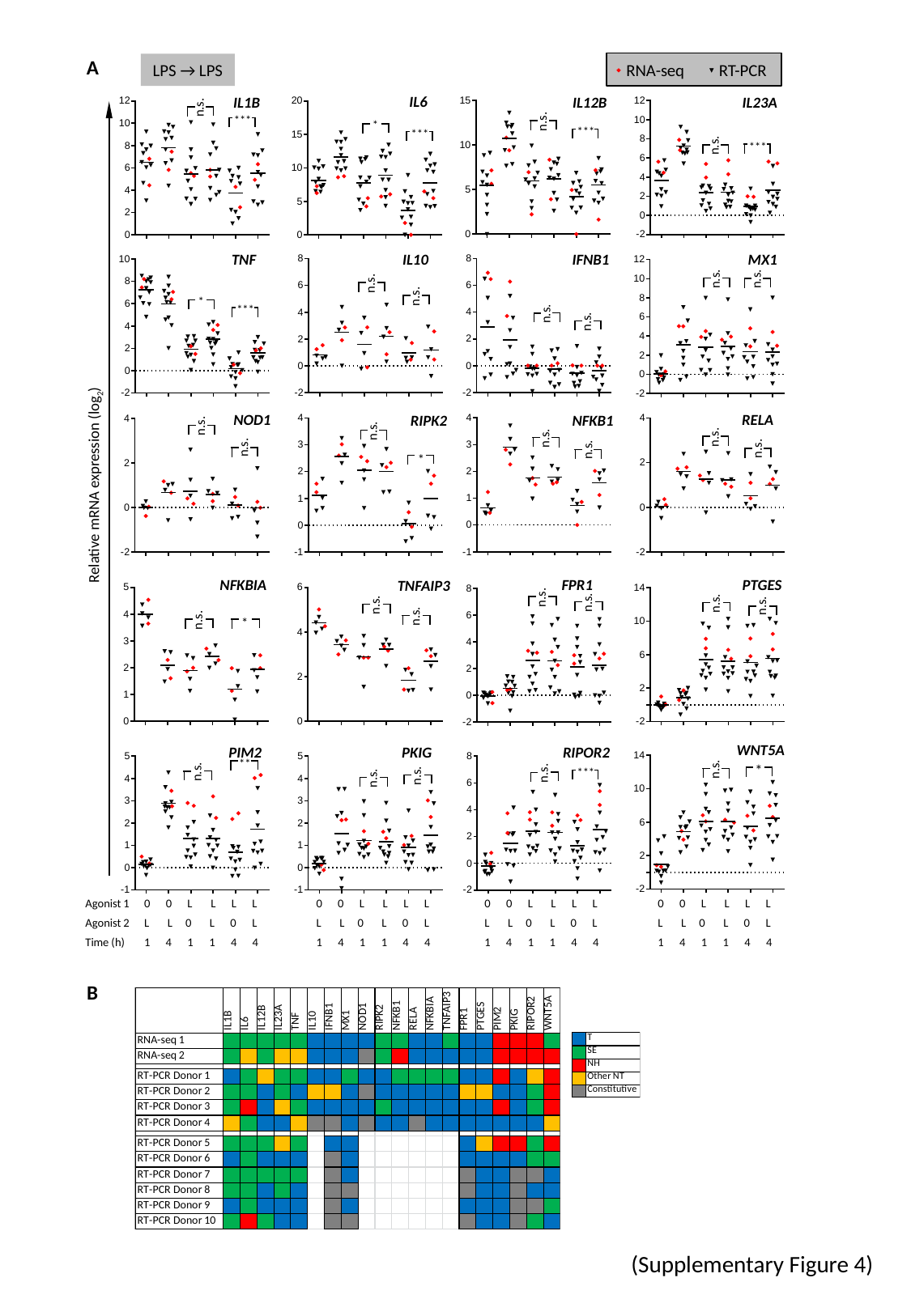

А
 RNA-seq RT-PCR
LPS → LPS
IL6
IL12B
IL23A
IL1B
n.s.
***
n.s.
*
***
***
n.s.
***
TNF
IL10
IFNB1
MX1
n.s.
n.s.
n.s.
n.s.
*
***
n.s.
n.s.
RELA
NOD1
NFKB1
RIPK2
n.s.
n.s.
n.s.
n.s.
n.s.
n.s.
n.s.
*
Relative mRNA expression (log2)
NFKBIA
FPR1
PTGES
TNFAIP3
n.s.
n.s.
n.s.
n.s.
n.s.
n.s.
n.s.
*
WNT5A
PIM2
PKIG
RIPOR2
**
n.s.
*
n.s.
***
n.s.
n.s.
n.s.
| Agonist 1 |
| --- |
| Agonist 2 |
| Time (h) |
| 0 0 L L L L |
| --- |
| L L 0 L 0 L |
| 1 4 1 1 4 4 |
| 0 0 L L L L |
| --- |
| L L 0 L 0 L |
| 1 4 1 1 4 4 |
| 0 0 L L L L |
| --- |
| L L 0 L 0 L |
| 1 4 1 1 4 4 |
| 0 0 L L L L |
| --- |
| L L 0 L 0 L |
| 1 4 1 1 4 4 |
B
(Supplementary Figure 4)

## Slide 6
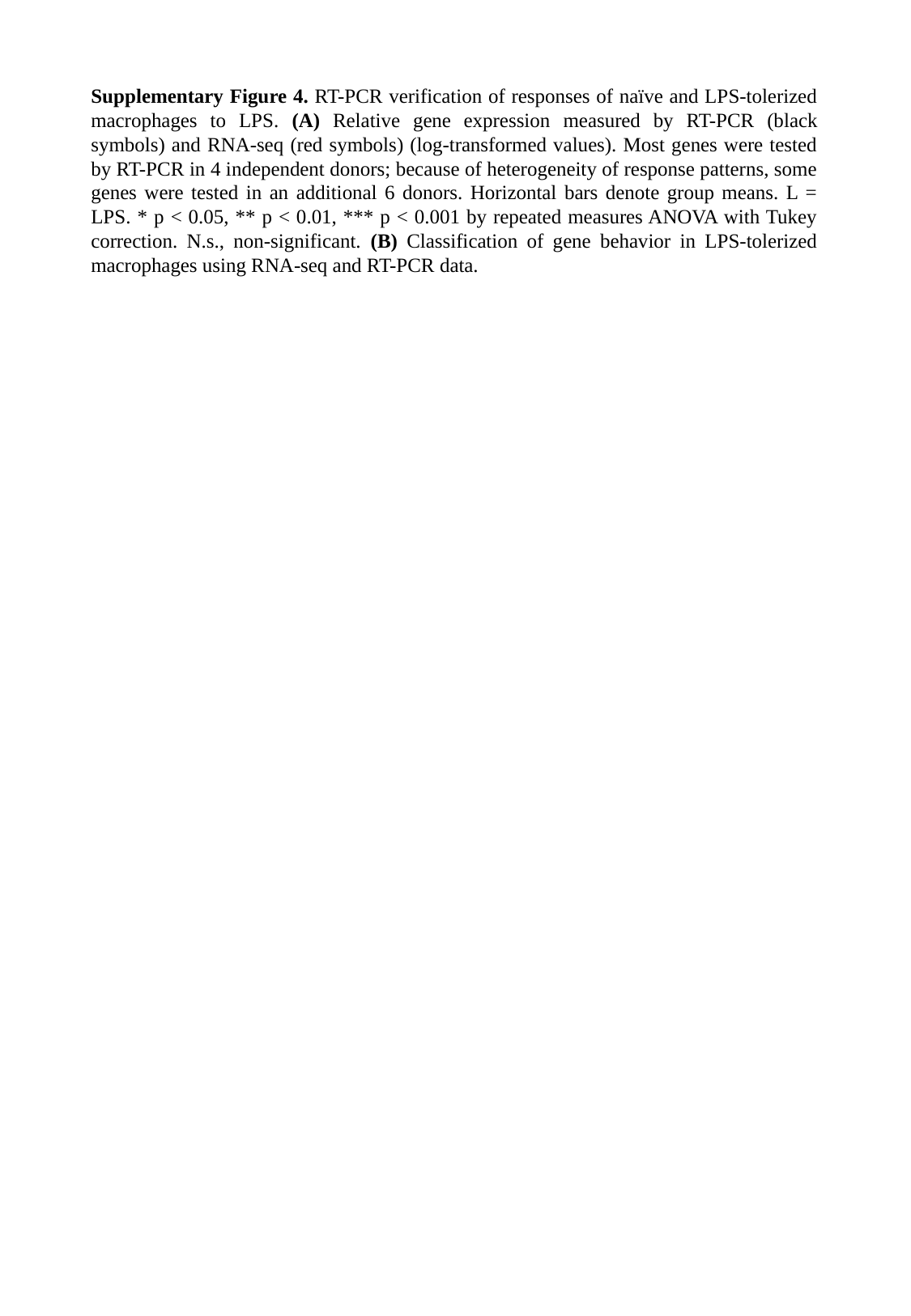

Supplementary Figure 4. RT-PCR verification of responses of naïve and LPS-tolerized macrophages to LPS. (A) Relative gene expression measured by RT-PCR (black symbols) and RNA-seq (red symbols) (log-transformed values). Most genes were tested by RT-PCR in 4 independent donors; because of heterogeneity of response patterns, some genes were tested in an additional 6 donors. Horizontal bars denote group means. L = LPS. * p < 0.05, ** p < 0.01, *** p < 0.001 by repeated measures ANOVA with Tukey correction. N.s., non-significant. (B) Classification of gene behavior in LPS-tolerized macrophages using RNA-seq and RT-PCR data.

## Slide 7
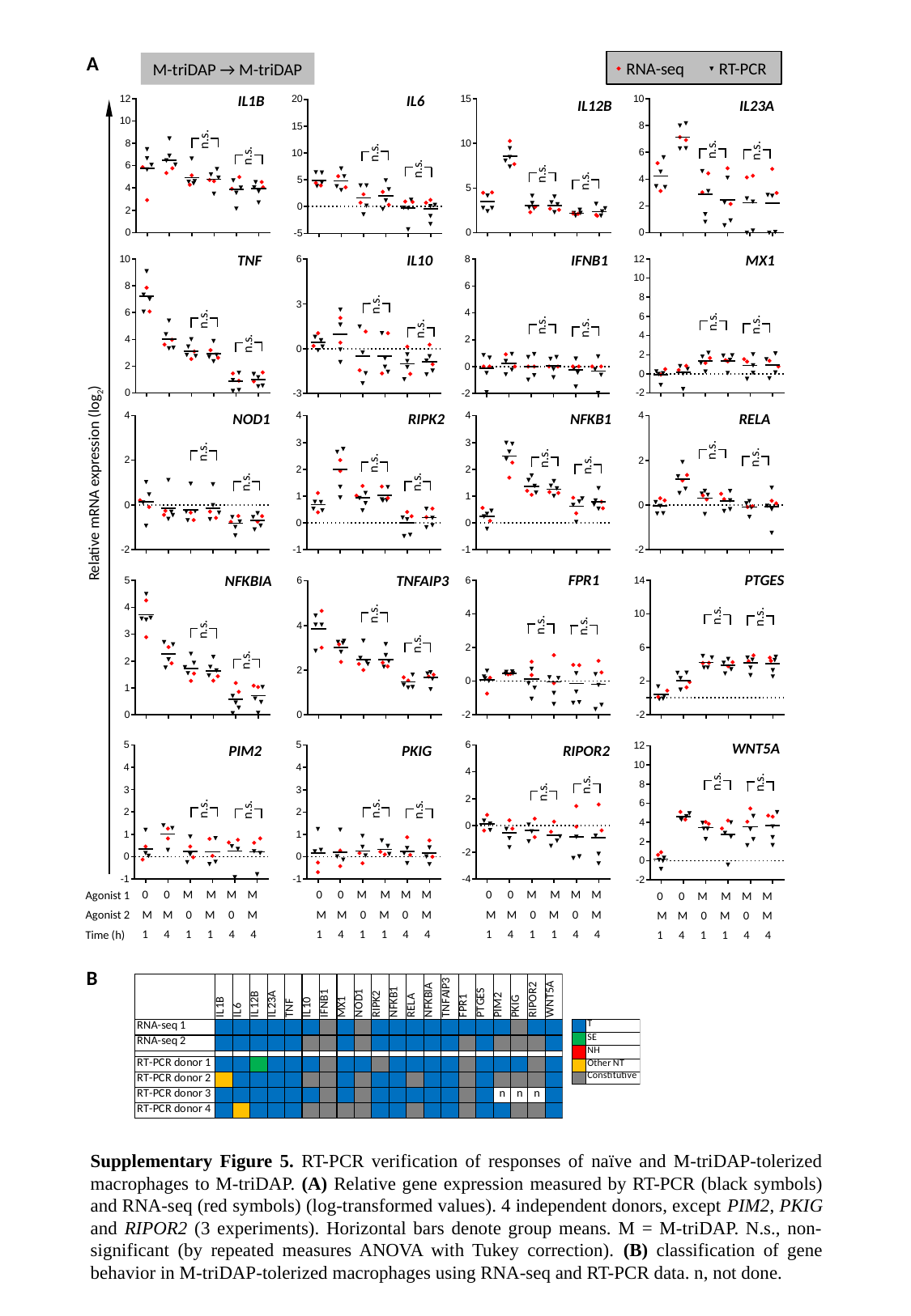

A
 RNA-seq RT-PCR
M-triDAP → M-triDAP
IL1B
IL6
IL12B
IL23A
n.s.
n.s.
n.s.
n.s.
n.s.
n.s.
n.s.
n.s.
IFNB1
MX1
TNF
IL10
n.s.
n.s.
n.s.
n.s.
n.s.
n.s.
n.s.
n.s.
NOD1
RIPK2
RELA
NFKB1
n.s.
n.s.
n.s.
n.s.
n.s.
n.s.
n.s.
n.s.
Relative mRNA expression (log2)
PTGES
FPR1
TNFAIP3
NFKBIA
n.s.
n.s.
n.s.
n.s.
n.s.
n.s.
n.s.
n.s.
WNT5A
PIM2
PKIG
RIPOR2
n.s.
n.s.
n.s.
n.s.
n.s.
n.s.
n.s.
n.s.
| 0 0 M M M M |
| --- |
| M M 0 M 0 M |
| 1 4 1 1 4 4 |
| 0 0 M M M M |
| --- |
| M M 0 M 0 M |
| 1 4 1 1 4 4 |
| 0 0 M M M M |
| --- |
| M M 0 M 0 M |
| 1 4 1 1 4 4 |
| Agonist 1 |
| --- |
| Agonist 2 |
| Time (h) |
| 0 0 M M M M |
| --- |
| M M 0 M 0 M |
| 1 4 1 1 4 4 |
B
Supplementary Figure 5. RT-PCR verification of responses of naïve and M-triDAP-tolerized macrophages to M-triDAP. (A) Relative gene expression measured by RT-PCR (black symbols) and RNA-seq (red symbols) (log-transformed values). 4 independent donors, except PIM2, PKIG and RIPOR2 (3 experiments). Horizontal bars denote group means. M = M-triDAP. N.s., non-significant (by repeated measures ANOVA with Tukey correction). (B) classification of gene behavior in M-triDAP-tolerized macrophages using RNA-seq and RT-PCR data. n, not done.

## Slide 8
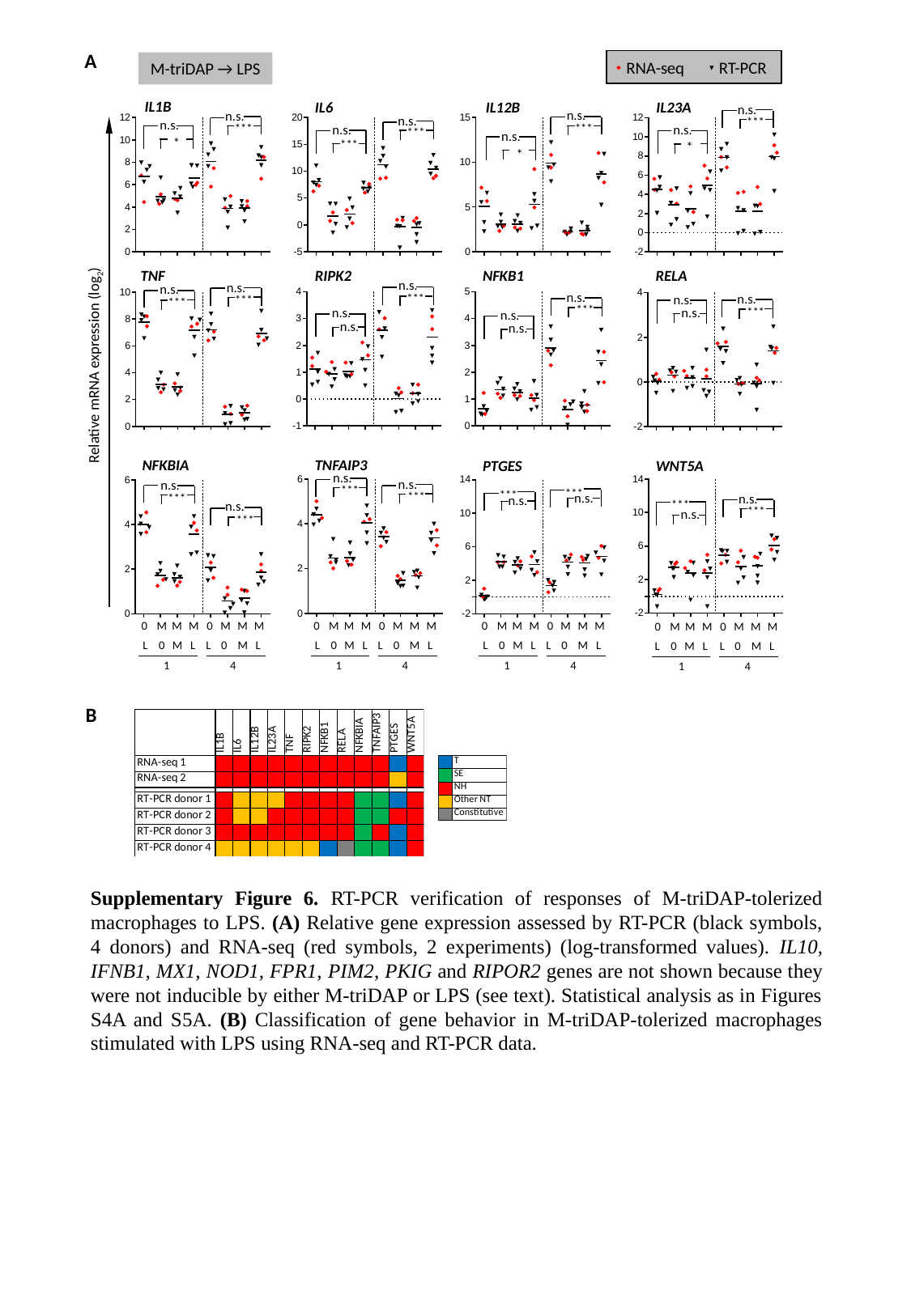

A
 RNA-seq RT-PCR
M-triDAP → LPS
IL1B
IL12B
IL23A
IL6
n.s.
n.s.
n.s.
n.s.
***
n.s.
***
***
n.s.
n.s.
***
n.s.
*
***
*
*
RIPK2
RELA
NFKB1
TNF
n.s.
n.s.
n.s.
n.s.
n.s.
***
n.s.
***
***
***
***
n.s.
n.s.
n.s.
n.s.
n.s.
Relative mRNA expression (log2)
TNFAIP3
NFKBIA
WNT5A
PTGES
n.s.
 n.s.
n.s.
***
***
***
***
n.s.
n.s.
***
n.s.
***
 n.s.
***
n.s.
***
| 0 M M M 0 M M M |
| --- |
| L 0 M L L 0 M L |
| 1 4 |
| 0 M M M 0 M M M |
| --- |
| L 0 M L L 0 M L |
| 1 4 |
| 0 M M M 0 M M M |
| --- |
| L 0 M L L 0 M L |
| 1 4 |
| 0 M M M 0 M M M |
| --- |
| L 0 M L L 0 M L |
| 1 4 |
B
Supplementary Figure 6. RT-PCR verification of responses of M-triDAP-tolerized macrophages to LPS. (A) Relative gene expression assessed by RT-PCR (black symbols, 4 donors) and RNA-seq (red symbols, 2 experiments) (log-transformed values). IL10, IFNB1, MX1, NOD1, FPR1, PIM2, PKIG and RIPOR2 genes are not shown because they were not inducible by either M-triDAP or LPS (see text). Statistical analysis as in Figures S4A and S5A. (B) Classification of gene behavior in M-triDAP-tolerized macrophages stimulated with LPS using RNA-seq and RT-PCR data.

## Slide 9
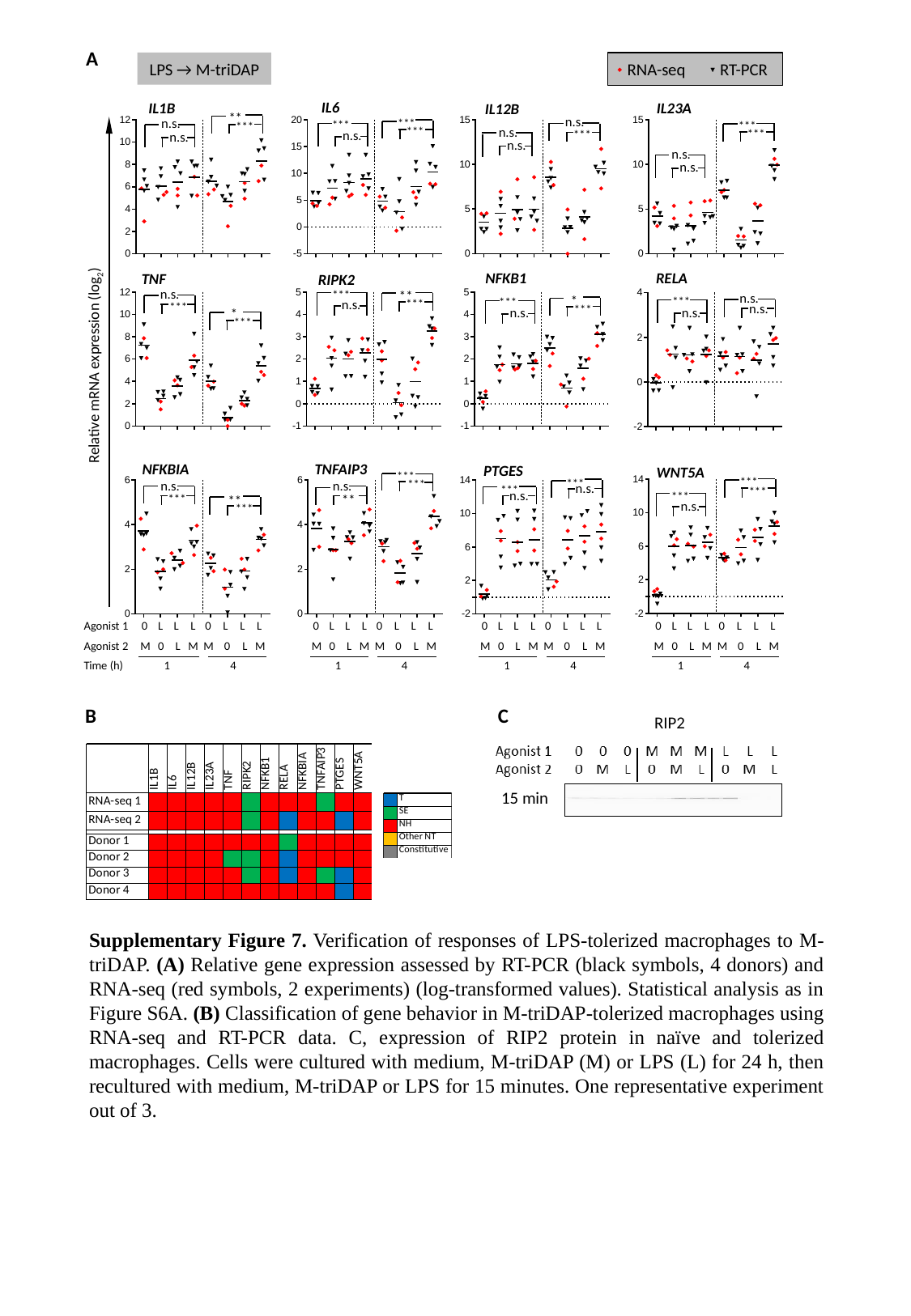

A
LPS → M-triDAP
 RNA-seq RT-PCR
IL6
IL23A
IL1B
IL12B
**
n.s.
n.s.
***
***
***
***
n.s.
***
***
***
n.s.
n.s.
n.s.
n.s.
n.s.
RELA
NFKB1
TNF
RIPK2
n.s.
**
***
n.s.
*
***
***
***
n.s.
***
n.s.
***
n.s.
n.s.
*
***
Relative mRNA expression (log2)
TNFAIP3
NFKBIA
PTGES
WNT5A
 ***
***
***
***
n.s.
n.s.
n.s.
***
***
n.s.
***
**
***
 **
n.s.
***
| Agonist 1 |
| --- |
| Agonist 2 |
| Time (h) |
| 0 L L L 0 L L L |
| --- |
| M 0 L M M 0 L M |
| 1 4 |
| 0 L L L 0 L L L |
| --- |
| M 0 L M M 0 L M |
| 1 4 |
| 0 L L L 0 L L L |
| --- |
| M 0 L M M 0 L M |
| 1 4 |
| 0 L L L 0 L L L |
| --- |
| M 0 L M M 0 L M |
| 1 4 |
C
B
RIP2
15 min
Supplementary Figure 7. Verification of responses of LPS-tolerized macrophages to M-triDAP. (A) Relative gene expression assessed by RT-PCR (black symbols, 4 donors) and RNA-seq (red symbols, 2 experiments) (log-transformed values). Statistical analysis as in Figure S6A. (B) Classification of gene behavior in M-triDAP-tolerized macrophages using RNA-seq and RT-PCR data. C, expression of RIP2 protein in naïve and tolerized macrophages. Cells were cultured with medium, M-triDAP (M) or LPS (L) for 24 h, then recultured with medium, M-triDAP or LPS for 15 minutes. One representative experiment out of 3.
